# Development of CRISPR-Cas13a-based antimicrobials capable of sequence-specific killing of target bacteria

**DOI:** 10.1038/s41467-020-16731-6

**Published:** 2020-06-10

**Authors:** Kotaro Kiga, Xin-Ee Tan, Rodrigo Ibarra-Chávez, Shinya Watanabe, Yoshifumi Aiba, Yusuke Sato’o, Feng-Yu Li, Teppei Sasahara, Bintao Cui, Moriyuki Kawauchi, Tanit Boonsiri, Kanate Thitiananpakorn, Yusuke Taki, Aa Haeruman Azam, Masato Suzuki, José R. Penadés, Longzhu Cui

**Affiliations:** 1 Division of Bacteriology, Department of Infection and Immunity, School of Medicine, Jichi Medical University, Tochigi, Japan; 2 Institute of Infection, Immunity & Inflammation, University of Glasgow, Glasgow, G12 8TA UK; 3 Antimicrobial Resistance Research Center, National Institute of Infectious Diseases, Tokyo, Japan

**Keywords:** Antimicrobials, Bacteriophages, Clinical microbiology, CRISPR-Cas systems, Infectious-disease diagnostics

## Abstract

The emergence of antimicrobial-resistant bacteria is an increasingly serious threat to global health, necessitating the development of innovative antimicrobials. Here we report the development of a series of CRISPR-Cas13a-based antibacterial nucleocapsids, termed CapsidCas13a(s), capable of sequence-specific killing of carbapenem-resistant *Escherichia coli* and methicillin-resistant *Staphylococcus aureus* by recognizing corresponding antimicrobial resistance genes. CapsidCas13a constructs are generated by packaging programmed CRISPR-Cas13a into a bacteriophage capsid to target antimicrobial resistance genes. Contrary to Cas9-based antimicrobials that lack bacterial killing capacity when the target genes are located on a plasmid, the CapsidCas13a(s) exhibit strong bacterial killing activities upon recognizing target genes regardless of their location. Moreover, we also demonstrate that the CapsidCas13a(s) can be applied to detect bacterial genes through gene-specific depletion of bacteria without employing nucleic acid manipulation and optical visualization devices. Our data underscore the potential of CapsidCas13a(s) as both therapeutic agents against antimicrobial-resistant bacteria and nonchemical agents for detection of bacterial genes.

## Abstract (web summary)

CRISPR technology is emerging as a potential antimicrobial against antimicrobial-resistant bacteria. Here the authors develop a bacteriophage delivered Cas13a system for killing target bacteria and detecting bacterial genes.

## Introduction

The emergence and spread of antimicrobial resistance among pathogenic bacteria has been a growing global public health concern for several decades^1^. According to the recent CDC’s report on antimicrobial resistance, >2.8 million antimicrobial-resistant infections occur in the U.S. each year, and >35,000 people die as a result^2^. It is also predicted that antimicrobial-resistant (AMR) pathogens will cause 10 million fatalities per year by 2050 if new antimicrobial strategies are not developed^3^. In fact, the emergence of AMR bacteria has led to the post-antibiotic era, in which many currently available antimicrobials are no longer effective. This is due in part to the decline in antibiotic innovation, as no new class of antibiotics has been developed against Gram-negative bacteria in >45 years, and only a limited number of antibiotics were in either phase II or III clinical trials^4^. Therefore, there is an urgent need for new strategies to develop alternative therapeutic approaches to prevent infections of AMR bacteria.

To this end, various nucleic acid-based antibacterials, peptides, bacteriophage therapies, antibodies, bacteriocins, and anti-virulence compounds have been recently developed^5^. Among these, CRISPR (clustered regularly interspaced short palindromic repeats)-Cas3- and Cas9-encoding phages provide a means to combat such threats by selectively killing AMR bacteria^6^. The CRISPR-Cas3 and CRISPR-Cas9 genome-editing constructs, which were designed to target AMR genes, were delivered into bacteria by packaging them into phages^7–11^ to achieve AMR gene-specific bacterial killing^7–12^ and prevent the spread of AMR genes^7^. However, because DNA cleavage of plasmid DNA does not result in bacterial death, at least not in the absence of other confounding variables, such as a toxin–antitoxin system, this strategy is ineffective in targeting bacteria with plasmid-borne AMR genes^9^.

CRISPR-Cas13a is a CRISPR-Cas type VI class 2 system and is characterized by RNA-guided single-stranded RNA (ssRNA) cleavage activity^13,14^. A 2016 study by Abudayyeh et al. demonstrated that CRISPR-Cas13a has promiscuous ssRNA cleavage activities and restricts host bacteria growth due to the degradation of the bacterial RNAs (ref. ^14^). This has turned out to be a defense system against phages^15^. When phages infect bacteria, CRISPR-Cas13a recognizes transcript of phage genome, which leads to nonspecific degradation of bacterial transcripts and arrests bacterial cell growth to prevent the spread of infection. Recently, we identified four different subtypes of CRISPR-Cas13a systems from 11 strains of six *Leptotrichia* species^16^. Among them CRISPR-Cas13a form *Leptotrichia shahii* (LshCas13a) had a significant inhibition effect on bacterial growth, consistent with the observation by Abudayyeh et al.^14^. We here report success in developing sequence-specific antimicrobials by packaging the LshCas13a into bacteriophage capsids, which can be used as both therapeutic agents against AMR bacterial infections and nonchemical agents to detect bacterial genes for diagnosis.

## Results

### Bactericidal activity of Cas13a

**Fig. 1:**
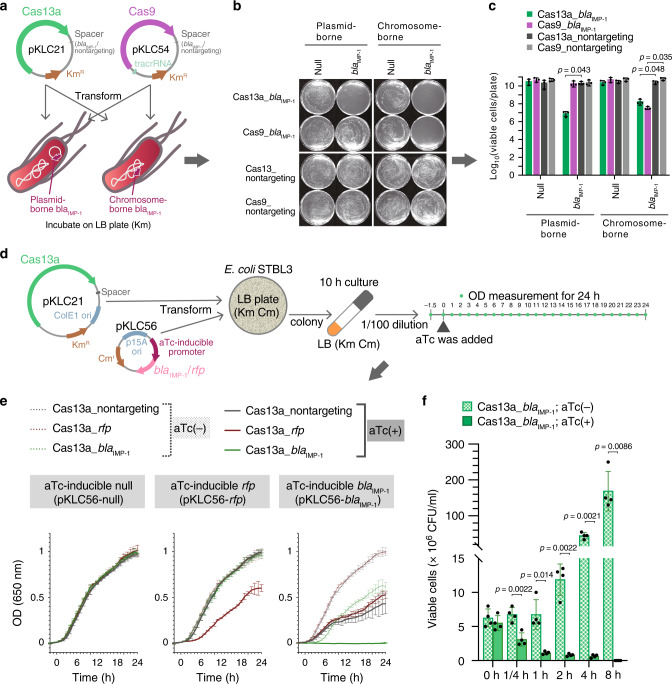
Sequence-specific bactericidal activity of CRISPR-Cas13a. **a** A schematic diagram of transformation of CRISPR-Cas13a and CRISPR-Cas9 with targeting *bla*_IMP-1_ into *bla*_IMP-1_-expressing *E. coli* STBL3. **b** *E. coli* STBL3 expressing *bla*_IMP-1_ from a plasmid (plasmid-borne *bla*_IMP-1_) and chromosome (chromosome-borne *bla*_IMP-1_) were prepared, and transformed with CRISPR-Cas13a or CRISPR-Cas9, both with spacer targeting *bla*_IMP-1_ or no spacer (nontargeting). The resulting transformants were plated on an LB plate containing kanamycin (Km) to test sequence-specific bacterial killing by CRISPR-Cas13a and CRISPR-Cas9. Km was used to maintain the plasmids. **c** The number of bacteria on the plate obtained in the experiment of **b** was counted. The statistical significance was determined by two-sided Student’s *t*-test. Each bar represents the mean with standard deviation (*n* = 3). **d** A schematic diagram of the experiment to test CRISPR-Cas13a-dependent cell growth inhibition. Anhydrotetracycline (aTc)-inducible *bla*_IMP-1_- or *rfp*-expression plasmid (pKLC56) was co-transformed with pKLC21 plasmid expressing CRISPR-Cas13a with spacer targeting *bla*_IMP-1_ or *rfp* into *E. coli* STBL3. The resulting transformants were then cultured and the aTc induction in the presence of the antibiotics Km and Cm (Km and Cm for maintaining plasmids) was carried out at the indicated time points. Thereafter, the OD values were measured every hour. **e** Growth curves were plotted. Each line of the growth curves represents the mean with standard deviation. **f** The number of viable cells was counted to calculate the ratio of cell death caused by *bla*_IMP-1_-targeting CRISPR-Cas13a. The bacterial culture prepared in **d** was diluted to 1/100, then aTc was added to induce *bla*_IMP-1_. Km and Cm were also added to maintain the CRISPR-Cas13a and the aTc-inducible *bla*_IMP-1_ plasmids. Thereafter, the bacterial cultures were sampled at the indicated time points, and the number of surviving bacteria was counted on fresh LB plates. Each bar represents the mean with standard deviation of four biological replicates. *p*-values were determined by two-sided Student’s *t*-test. Source data are available in the Source Data file.

First, we verified the growth inhibition ability of CRISPR-Cas13a (LshCas13a) in comparison with CRISPR-Cas9 by using *Escherichia coli* carrying the carbapenem resistance gene *bla*_IMP-1_ on a chromosome or plasmid. To this end, we constructed two plasmids (pKLC21 and pKLC54) harboring CRISPR-Cas13a or CRISPR-Cas9 with spacers targeting *bla*_IMP-1_ (spacer sequence was optimized, see later), and introduced them into the *E. coli*, respectively, to test their growth inhibition effect against the host cells carrying *bla*_IMP-1_ (Fig. 1a). As expected, the LshCas13a with targeting *bla*_IMP-1_ decreased the number of bacteria by two to three orders of magnitude against the bacteria carrying *bla*_IMP-1_ both on chromosome (from 2.6 × 10^10^ to 2.0 × 10^8^ CFU/ml) and plasmid (from 2.3 × 10^10^ to 8.7 × 10^6^ CFU/ml). However the CRISPR-Cas9 decreased the number of bacteria by three orders of magnitude against the bacteria carrying the *bla*_IMP-1_ only on the chromosome (from 6.3 × 10^10^ to 3.6 × 10^7^ CFU/ml), but not on the plasmid (from 2.8 × 10^10^ to 2.2 × 10^10^ CFU/ml), when compared with their respective nontargeting controls (Fig. 1b, c). These results agreed with the fact that while CRISPR-Cas9 caused cell death by double-strand DNA breaks, CRISPR-Cas13a induced cell dormancy by collateral nonspecific cleavage of RNA (ref. ^14^). However, it is not clear whether the above decrements in cell number by Cas13a is due to cell death.

In order to determine whether Cas13a causes cell death or not, we have constructed a system in which CRISPR-Cas13a targeting mRNA of red fluorescent protein (RFP) transcribed from plasmid inhibits the growth of bacteria harboring RFP, following the method by Abudayyeh et al.^14^. We carried out the experiment in broth culture medium using LshCas13a by introducing an anhydrotetracycline (aTc)-inducible RFP plasmid (pKLC56-*rfp*), as well as an RFP-targeting LshCas13a (Cas13a_*rfp*) into *E. coli* (Fig. 1d), where the same spacer sequence used by Abudayyeh et al. was used to target *rfp* (ref. ^14^). We observed the similar cell growth restriction by LshCas13a upon induction of the target *rfp* transcription compared to nontarget control and non-induction control (Fig. 1e, left and middle panels). Interestingly, when the same experiment was carried out using *bla*_IMP-1_ as target gene, the bacteria growth curve stopped rising upon induction of *bla*_IMP-1_ transcription (Fig. 1e, right panel), indicating cell growth might be completely stopped due to the cell death or dormancy. To make this point clear, viable cells carrying *bla*_IMP-1_-targeting spacer were counted during the culture with and without aTc induction of *bla*_IMP-1_ transcription. As seen in Fig. 1f, the number of viable cells were decreased by about three orders of magnitude from 5.5 × 10^6^ to 8.8 × 10^3^ during the 8 h upon induction of *bla*_IMP-1_ transcription. We interpreted these results as demonstration of CRISPR-Cas13a-mediated sequence-specific killing of host cells. However, reasons of the viable cells that are in dormancy^15^ or resistant to CRISPR-Cas13a are not clear at the moment, but we did not find any mutation in target sequence after sequencing 32 randomly selected colonies from the above two experiments (Fig. 1b). We also observed that the induction of *bla*_IMP-1_ transcription inhibited the host cell growth (Fig. 1e, right panel), which could be attributed to fitness cost of *bla*_IMP-1_ overexpression. It is well known that the accumulation of carbapenemase compromises the bacterial growth^17,18^.

### Packaging of CRISPR-Cas13a into bacteriophage capsid

**Fig. 2:**
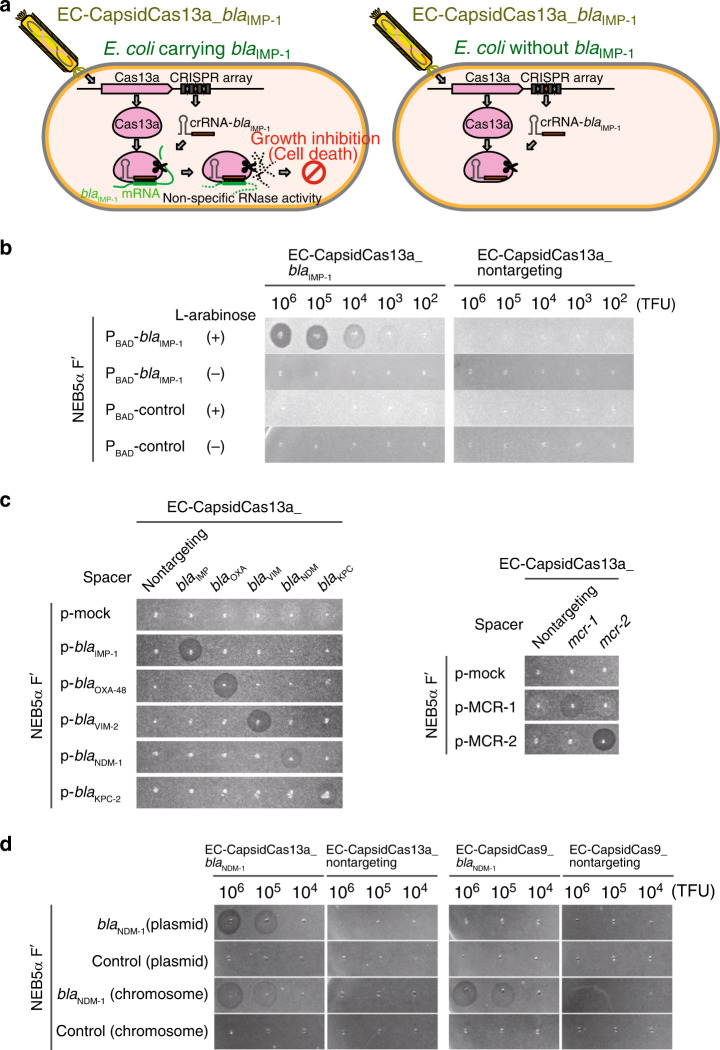
In vivo sequence-specific bactericidal activity of CapsidCas13a. **a** Graphical concept model depicting the mode of action of CapsidCas13a, with *bla*_IMP-1_-targeting CRISPR-Cas13a packaged into *E. coli* M13 phage capsid (EC-CapsidCas13a-*bla*_IMP-1_) for delivery into host bacterial cells during the normal course of viral infection. In the presence of the target gene (*E. coli* carrying *bla*_IMP-1_), CRISPR-Cas13a will be activated and the subsequent non-sequence-specific RNase activity will result in host cell death. In the absence of the target gene (*E. coli* without *bla*_IMP-1_), CRISPR-Cas13a will not be activated and there will be no subsequent cell death. **b**–**d** Spot assay with generated CapsidCas13a(s) on bacterial lawn of *E. coli* NEB5α F′*I*^q^ to test bactericidal activity. A clear lysis zone of the spotted area represents bacterial killing. NEB5α F′*I*^q^ carrying *bla*_IMP-1_ expression vector and empty vector (control) were infected with tenfold serial dilutions of EC-CapsidCas13a-*bla*_IMP-1_ in the presence or absence of L-arabinose for inducing *bla*_IMP-1_ expression (**b**); CapsidCas13a(s) programmed to target different carbapenem resistance genes (*bla*_IMP-1_, *bla*_OXA-48_, *bla*_VIM-2_, *bla*_NDM-1_, and *bla*_KPC-2_) and colistin resistance genes (*mcr-1* and *mcr-2*) were spotted on a series of NEB5α F′*I*^q^ strains expressing each of the resistance genes (**c**); comparison of bactericidal activity between CRISPR-Cas9 and CRISPR-Cas13a. Nontargeting and *bla*_NDM-1_-targeting CapsidCas13a and CapsidCas9, in tenfold serial dilutions, were spotted on lawn of NEB5α F′*I*^q^ with or without expression of *bla*_NDM-1_ on plasmid or chromosome (**d**). All assays were replicated three times.

Having verified the bactericidal activity of the CRISPR-Cas13a_*bla*_IMP-1_ construct against *bla*_IMP-1_-positive bacteria, we came up with the idea that AMR bacteria-specific CapsidCas13a constructs could be synthesized by loading the CRISPR-Cas13a system into a phage capsid. The CRISPR-Cas13a_*bla*_IMP-1_ was loaded into *E. coli* phage M13 capsid, to generate EC-CapsidCas13a_*bla*_IMP-1_ (Fig. 2a), which demonstrated sequence-specific killing activity against bacteria carrying the *bla*_IMP-1_ gene in an EC-CapsidCas13a_*bla*_IMP-1_ concentration-dependent manner (Fig. 2b). Subsequently, we confirmed the same sequence-specific killing activities with a series of CapsidCas13a constructs programmed to target different genotypes of carbapenem-resistant genes (*bla*_IMP-1_, *bla*_OXA-48_, *bla*_VIM-2_, *bla*_NDM-1_, and *bla*_KPC-2_) and colistin-resistant genes (*mcr*-1 and *mcr*-2), all of which are currently a problem in the clinical setting (Fig. 2c, Supplementary Fig. 1). Conversely, nontargeting CapsidCas13a (with nontargeting spacer) killed no bacteria under any circumstance, indicating that this construct has robust specificity for gene-directed antimicrobial therapy against AMR bacterial infections.

In order to compare the characteristics of CapsidCas13a with previously reported Cas9-based antimicrobial agents, we generated a Cas9-based EC-CapsidCas9_*bla*_NDM-1_ construct with the same protocol, but in this instance CRISPR-Cas13a_*bla*_NDM-1_ was replaced with CRISPR-Cas9_*bla*_NDM-1_ and its bactericidal manner was compared with that of EC-CapsidCas13a_*bla*_NDM-1_. As expected, EC-CapsidCas9_*bla*_NDM-1_ killed only the bacteria with a chromosomal target gene, while EC-CapsidCas13a_*bla*_NDM-1_ killed the bacteria that carried the target gene on either the chromosome or plasmid (Fig. 2d). Since many clinically important AMR genes, including carbapenem-resistant genes found in Enterobacteriaceae are encoded on plasmids^19,20^, CapsidCas13a was expected to be superior to CapsidCas9 in light of the killing efficiency. Besides, previous studies have suggested that CRISPR-Cas9 is prone to unexpected genetic mutations due to its DNA cleavage activity^21–23^. On the contrary, instead of the direct cleavage of DNA, CapsidCas13a targets bacterial mRNA. Therefore, unexpected genetic mutations of the bacteria are less likely to occur due to the action of Cas13a. Although an intensive study is needed, at least, as mentioned above we did not find any mutation in target genes by sequencing 32 Cas13a-resistant colonies. These characteristics showed a different potential of Cas13a as an antimicrobial agent compared with Cas9.

**Fig. 3:**
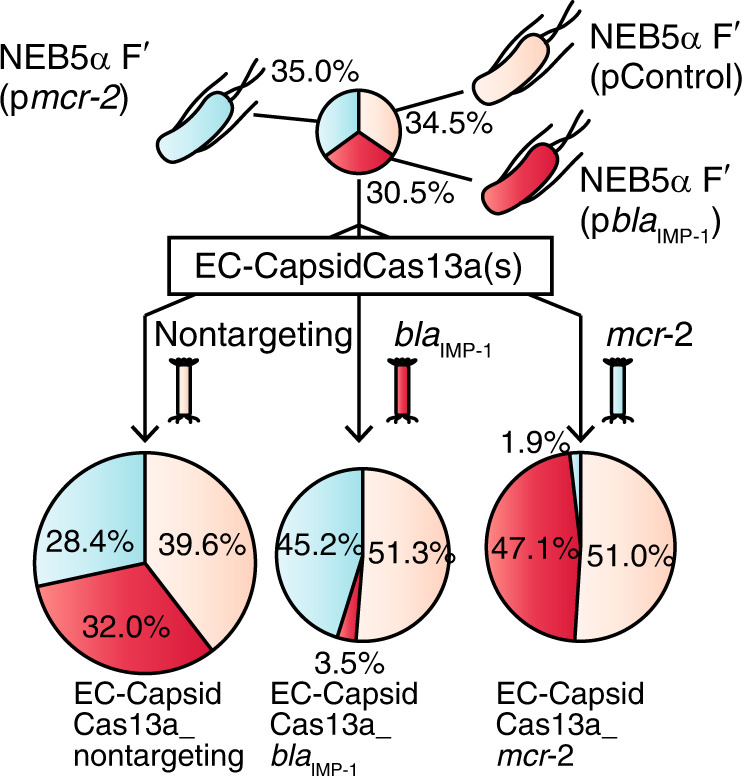
Potential of CapsidCas13a as a tool for modifying bacterial flora. Programmed CapsidCas13a altered the composition of bacterial population. A mixed cell population was prepared by mixing *E. coli* NEB5α F′*I*^q^ (control) with equal numbers of NEB5α F′*I*^q^ expressing *bla*_IMP-1_ and *mcr-2*, respectively. The cell mixtures were then independently treated with *bla*_IMP-1_-targeting, *mcr-2*-targeting, and nontargeting CapsidCas13a. Note that each AMR gene-targeting CapsidCas13a reduced the corresponding target cell population. The percentage represents the mean of three biological replicates. Source data are available in the Source Data file.

**Fig. 4:**
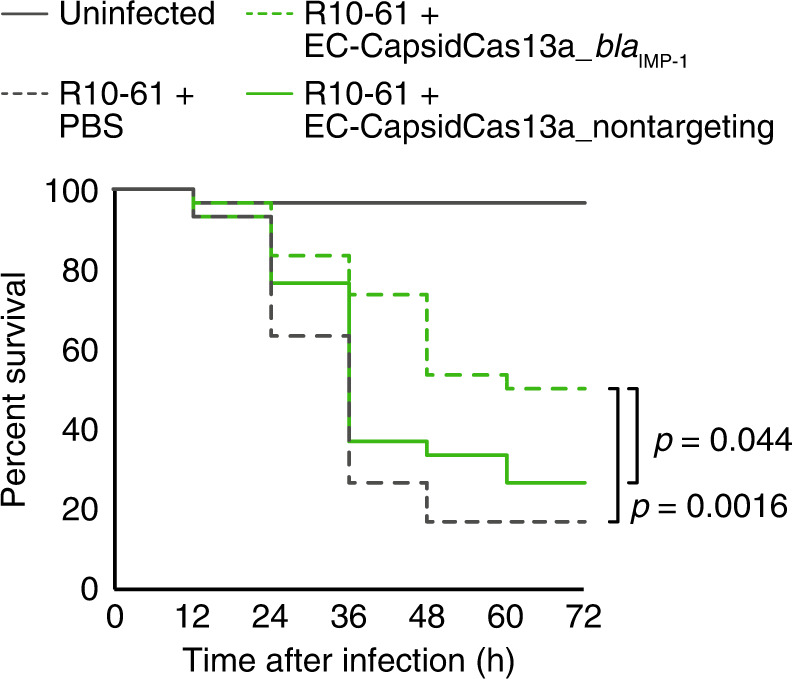
Potential of CapsidCas13a as a therapeutic against AMR bacteria infections. Examination of the therapeutic effect of EC-CapsidCas13a-*bla*_IMP-1_ using a *Galleria mellonella* infection model. Administration of EC-CapsidCas13a-*bla*_IMP-1_ (MOI 100) into *G. mellonella* larvae infected with R10-61 (carbapenem-resistant clinical isolates of *E. coli* carrying *bla*_IMP-1_) significantly improved host survival compared to controls, EC-CapsidCas13a-nontargeting (*p* = 0.044), and phosphate-buffered saline (PBS; *p* = 0.0016). The *p*-value between two groups infected with R10-61, and treated with EC-CapsidCas13a-nontargeting and PBS was 0.30. The *p*-values are calculated by log-rank test. The results are presented as the aggregate values of three independent experiments performed using ten larvae per group. Source data are available in the Source Data file.

In order to determine whether the CapsidCas13a(s) can selectively kill target bacteria among a mixed population of AMR bacteria, an artificial mixture of *E. coli* NEB5α F′*I*^*q*^ expressing the plasmid-borne carbapenem resistance gene *bla*_IMP-1_, the colistin resistance gene *mcr-2*, or with no resistance gene as a control, was treated with single-dose of each gene-specific EC-CapsidCas13a construct (i.e., EC-CapsidCas13a_*bla*_IMP-1_ for targeting *bla*_IMP-1_, and EC-CapsidCas13a_*mcr-2* for targeting *mcr-2*). Subsequently, the abundance of each bacterial strain was determined. As shown in Fig. 3, the percentage of cell numbers of NEB5α F′*I*^*q*^ (*bla*_IMP-1_) and NEB5α F′*I*^*q*^ (*mcr-2*) decreased significantly from 30.5% and 35.0% to 3.5% and 1.9%, respectively when the cell mixtures were treated with EC-CapsidCas13a_*bla*_IMP-1_ and EC-CapsidCas13a_*mcr-2*, respectively. Meanwhile there was no change in the abundance of cell populations after being treated by EC-CapsidCas13a with nontargeting spacer control. These sequence-specific killing activities of CapsidCas13a(s) demonstrate their potential as microbial control agents that can modify or manipulate the bacterial flora without affecting undesired bacterial populations by killing those with specific genomic contents. Besides demonstrating of the versatility of CapsidCas13a, we investigated its in vivo therapeutic efficacy using a *Galleria mellonella* larvae infection model. Administration of EC-CapsidCas13a_*bla*_IMP-1_ to *G. mellonella* larvae infected with *E. coli* R10-61 expressing *bla*_IMP-1_ showed significantly improved survival over no treatment (*p* = 0.0016) or a CapsidCas13a carrying nontargeting spacer, as a control (*p* = 0.044) (Fig. 4). This outcome further strengthened the therapeutic prospects of the CapsidCas13a constructs for treatment of infections with AMR bacteria.

### Application to bacterial gene detection

**Fig. 5:**
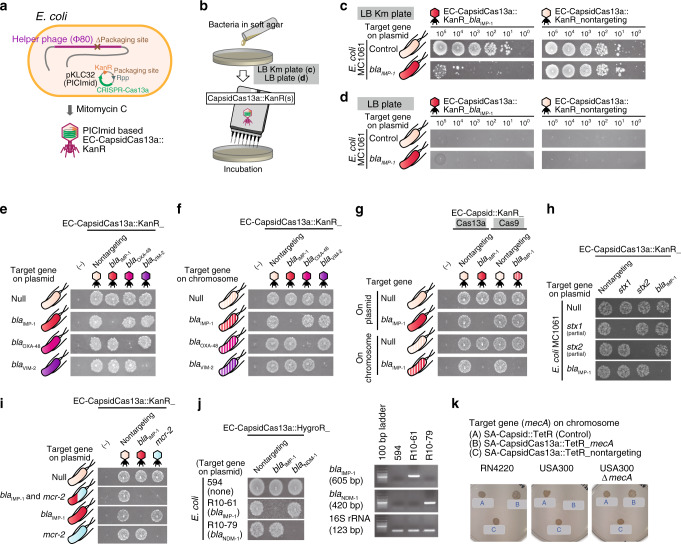
Potential use of CapsidCas13a for bacterial gene detection. **a** A schematic illustration of generation of PICI-based EC-CapsidCas13a targeting *bla*_IMP-1_. Mitomycin C induction promotes the packaging of PICImid carrying a CRISPR-Cas13a system and kanamycin (Km) resistance gene into the capsid of helper phage Φ80. **b**–**d** Bactericidal activity test of PICI-based EC-CapsidCas13a::KanR-*bla*_IMP-1_ (in tenfold serial dilutions) against *E. coli* MC1061 with or without the expression of target gene was carried out on LB agar plates (**b**); and the test results were judged by observing bacterial growth on LB bottom agar plates supplemented with Km (**c**), or observation of cell lysis on drug-free LB bottom agar plates (**d**); noting that the former assay had an enhanced sensitivity by about three orders of magnitude against the bacteria carrying target gene. **e**–**j** The PICI-based EC-CapsidCas13a(s) were applicable to detect various carbapenem resistance genes (*bla*_IMP-1_, *bla*_OXA-48_, and *bla*_VIM-2_) regardless of their location on either the plasmid or chromosome (**e**, **f**), whereas EC-CapsidCas9 could detect genes located on the chromosome but not on the plasmid (**g**); and the PICI-based EC-CapsidCas13a(s) also effectively detected toxin-encoded genes (**h**), differentiated different genes located on the same plasmid (**i**), and were also applicable to clinical isolates (**j**, left panel) as being verified by PCR (**j**, right panel). **k** SA-CapsidCas13a::TetR-*mecA* generated by packaging *mecA*-targeting CRISPR-Cas13a into capsid of *S. aureus* phage 80α exhibited *mecA*-specific bactericidal activity against MRSA, but not *S. aureus* strains deficient in *mecA*. All assays were replicated three times. Uncropped images of the gels are available in the Supplementary Information.

Because the prevalence of stealth bacteria (carrying AMR genes but not identifiable by existing antibiotic susceptibility tests) has been increasing^24^, a simple and easy-to-use detection method for such strains is required in the clinical setting. To this end, the CapsidCas13a constructs were modified for the detection of bacterial AMR genes. First, the spacer sequence of EC-CapsidCas13a_*bla*_IMP-1_ targeting the *bla*_IMP-1_ gene was optimized in order to improve the killing efficiency. The optimization started with the construction of a CRISPR-Cas13a expression plasmid library, in which 121 spacer sequences targeting different positions throughout the whole *bla*_IMP-1_ were inserted, each as one, into the CRISPR array. Then, all plasmids were individually transformed into *bla*_IMP-1_-expressing *E. coli* cells and the resulting transformants were analyzed to identify the most effective spacer sequence (Supplementary Fig. 2a). The calculation of the number of spacer reads after deep sequencing of the total plasmid DNAs extracted from the transformants found that all of the tested spacer sequences mediated target-specific bacterial killing, at least to some extent, when judged by the depletion rate of the plasmid DNAs (Supplementary Fig. 2b). The depletion rate was calculated by normalizing the number of reads from cells expressing *bla*_IMP-1_ with that of non-*bla*_IMP-1_-_-_expression. Among these, 13 spacer sequences had depletion rates of >99.0 (Supplementary Fig. 2c). The *bla*_IMP-1__563 spacer sequence GACTTTGGCCAAGCTTCTATATTTGCGT, which had the highest depletion rate of 99.7, was chosen as the best spacer sequence for use in subsequent experiments (Supplementary Fig. 2c, Supplementary Table 4). Then, the carrier M13 phage was replaced with the lysogenic phage Φ80 for use with the phage-inducible chromosomal island (PICI) packaging system (Supplementary Fig. 3)^25,26^, which is more flexible in genome manipulation. Finally, the kanamycin (Km) resistance gene (KanR) was inserted as a selection marker to generate the constructs of PICI-based EC-CapsidCas13a::KanR_*bla*_IMP-1_ and EC-CapsidCas13a::KanR_nontargeting as a nontargeting spacer control (Fig. 5a).

Next, we tested the detection efficiency of the constructs for *bla*_IMP-1_ by spotting 2 µL of tenfold serial dilutions of EC-CapsidCas13a::KanR_*bla*_IMP-1_ and EC-CapsidCas13a::KanR_nontargeting onto fresh top agar lawns of the test strains in Luria-Bertani (LB) agar with or without supplementation of Km (Fig. 5b). To improve the efficiency, we opted to determine the bacterial killing effect against Km-resistant cells on Km plates (Fig. 5c), rather than assessing the bacterial killing effect against original cells on Km-free plates (Fig. 5d). When the EC-CapsidCas13a::KanR_*bla*_IMP-1_ carrying the Km resistance gene was applied on the soft agar bacterial lawn grown on bottom agar containing Km, the cells infected by this capsid acquired Km resistance and, hence, could grow in the presence of Km. Nevertheless, with the bacterial cells carrying the target AMR gene *bla*_IMP-1_, there was no observable growth due to the bactericidal effect of the CRISPR-Cas13a construct (Fig. 5c). This method was shown to be almost three orders of magnitude more sensitive than direct observation of the bacterial growth inhibition on Km-free plates (Fig. 5d). We further confirmed the efficiency of this system, with the use of the M13 capsid-based EC-CapsidM13Cas13a::KanR_*bla*_IMP-1_ construct (Supplementary Fig. 4a–c). A subsequent experiment showed that the target AMR genes of interest located on either the plasmid or chromosome could be precisely detected (Fig. 5e, f, Supplementary Fig. 4d, e), as expected, whereas CRISPR-Cas9 construct could detect only the genes located on a chromosome but not on a plasmid (Fig. 5g). The detection ability was further confirmed with the CapsidCas13a constructs targeting other AMR genes *bla*_OXA-48_ and *bla*_VIM-2_ (Fig. 5e, f), toxin genes *stx*1 and *stx*2 (Fig. 5h), and two genes (*bla*_IMP-1_ and *mcr-2*) located on the same plasmid (Fig. 5i), indicating that this method is applicable for the detection of any bacterial genes regardless of their location on bacterial chromosome or plasmid. Although the sensitivity was slightly lower, it was even possible to detect target genes by directly spotting the CapsidCas13a(s) onto the bacteria swabbed on an agar plate instead of using the soft agar overlay method (Supplementary Fig. 5).

We also tried to apply the CapsidCas13a constructs to detect carbapenem-resistant clinical isolates of *E. coli* carrying *bla*_IMP-1_ or *bla*_NDM-1_. As these strains were not susceptible to Km, the KanR of EC-CapsidCas13a::KanR was replaced with the hygromycin (Hygro) resistance gene, HygroR, to generate the constructs EC-CapsidCas13a::HygroR_*bla*_IMP-1_ and EC-CapsidCas13a::HygroR_*bla*_NDM-1_. The test results with these two CapsidCas13a(s) showed that the *E. coli* clinical isolates carrying *bla*_IMP-1_ and *bla*_NDM-1_ were precisely detected, which was consistent with the results of polymerase chain reaction (PCR) analysis (Fig. 5j, Supplementary Fig. 5d), suggesting that the CapsidCas13a(s) can be applied to detect bacterial genes. With regard to the application potential of CRISPR-Cas13a, our data demonstrated above has opened up a new field in which it can be developed not only as an antibacterial therapeutic agent, but also as a new bacterial identification system where no nucleic acid manipulation is necessary once the CapsidCas13a(s) are established. There was an elegant report on the CRISPR-Cas13-based nucleic acid detection method, called as SHERLOCK system, which was developed by combination of nucleic acid amplification technique and CRISPR-Cas13. This method can detect DNA or RNA in vitro with attomolar level sensitivity^27^. In contrast, the proposed detection system using the CapsidCas13a constructs can be performed without amplification of DNA or RNA, electrophoresis equipment or optical devices, as only bacterial culture plates are required. In addition, >10^10^ transducing forming units (TFU) of CapsidCas13a constructs can be harvested per liter of host bacterial culture, and only 2–3 µL of 10^5^ constructs per mL of solution is required for a single spot test to accurately determine the presence or absence of target genes. Although these features highlight the elegant potential of CapsidCas13a(s) for bacterial gene detection, there are still limitations, at least: (1) it is necessary to construct corresponding CapsidCas13a for each bacterial species and gene, (2) turnaround time for test results can be long since interpretation of the results is dependent on bacterial growth, (3) it cannot be used when the bacteria cannot be cultured or the target gene is not transcribed.

### *Staphylococcus aureus* CapsidCas13a

Lastly, in addition to demonstrating the bactericidal activity of CRISPR-Cas13a against Gram-negative bacteria, we also attempted to confirm the collateral activity of CRISPR-Cas13a against Gram-positive bacteria using *Staphylococcus aureus*. First, a set of *E. coli–S. aureus* shuttle vectors, namely pKLC4(s), were generated carrying the CRISPR-Cas13a construct with or without a spacer sequence targeting the *S*. *aureus rpsE* genes. Transformation of the vector into *S. aureus* strain RN4220 showed that the bactericidal activity of CRISPR-Cas13a with an appropriate spacer sequence was similar to that in *E. coli* (Supplementary Fig. 6a). Then, a SA-CapsidCas13a_*mecA* construct was produced to target methicillin-resistant gene *mecA* of methicillin-resistant *S. aureus* (MRSA), one of the most prevalent AMR pathogens worldwide^28^. We optimized the spacer sequences (Supplementary Fig. 6b–e) and the CRISPR-Cas13a_*mecA*-carrying vector (pKLC-SP_*mecA*) construction were carried out in the same way as above for CRISPR-Cas13a_*bla*_IMP-1_. The packing of CRISPR-Cas13a_*mecA* into *S. aureus* phage 80α capsid was performed in accordance with the method established by Ubeda et al. using the *S. aureus* pathogenicity island (SaPI) system^29,30^ (Supplementary Fig. 7). This packing system simultaneously imparted tetracycline (Tet) resistance to the resulting SA-CapsidCas13a::TetR_*mecA* construct, since the SaPI carried the Tet resistance gene, which made it possible to be tested for both bactericidal ability against MRSA and capability of MRSA detection by targeting *mecA*. As expected, when the methicillin-susceptible *S. aureus* strain RN4220 and MRSA USA300 or *mecA* knockout USA300 (USA300-Δ*mecA*) were infected with SA-CapsidCas13a_*mecA*, the growth of only the USA300 strain carrying *mecA* was significantly inhibited (Fig. 5k, Supplementary Fig. 8), clearly demonstrating the sequence-specific bacterial killing ability of CRISPR-Cas13a against the Gram-positive bacteria *S. aureus*.

## Discussion

In this study, we employed the promiscuous RNA cleavage ability of CRISPR-Cas13a via recognition of target RNA by CRISPR-RNA (crRNA), which resulted in host cell death, to generate a new type of sequence-specific bacterial antimicrobials. To deliver the CRISPR-Cas13a to target bacteria, we packaged the CRISPR-Cas13a into carrier phage capsid using the PICI packaging system for *E. coli*, and SaPI packaging system for *S. aureus*. Since synthesized CapsidCas13a does not carry phage genome, it thus belongs to the category of a nucleic acid drug or gene drug, not an organism, thereby easily being put into practical use as a therapeutic drug. Although there are still many questions to be answered concerning practical application—such as host range of the phage capsids, catalytic mode of Cas13a (refs. ^14,15^), the efficiency of phage capsid packaging, and ethical issues regarding genetic recombinants, etc.—our strategy demonstrated that the CapsidCas13a antimicrobials are promising to be developed for at least three application categories: (1) as promising antibacterial therapeutic agents targeting any bacterial gene, including AMR genes, or selectively killing targeted toxin-producing bacteria, (2) as a simple and inexpensive bacterial gene detection system for bacterial identification and efficient molecular epidemiological investigations without the need for the amplification of nucleic acids or optic devices, (3) as tools to manipulate the bacterial flora by targeting and eliminating a specific bacterial population without disrupting other irrelevant bacterial populations. In conclusion, the proposed CRISPR-Cas13a-based antimicrobials are expected to have a great impact in the field of antimicrobial resistance for infection control, as well as bacterial flora control.

## Methods

### Ethics declarations

Ethics approval for the use of invertebrates was given by Jichi Medical University ethics committee.

### Bacterial strains and culture conditions

Bacterial strains used in this study are listed in Supplementary Table 1. Bacterial strains were grown at 37 °C in LB medium (BD Difco). Unless otherwise indicated, proper antibiotics were added to growth medium to the following final concentrations: 100 µg/mL for ampicillin (Amp), 30 µg/mL for Km, 34 µg/mL for chloramphenicol (Cm), 4 µg/mL for colistin, and 200 µg/mL for Hygro.

### CRISPR-Cas13a and CRISPR-Cas9 gene targeting vectors

The CRISPR-Cas13a expression vector (pC003), which carries LshC2c2 locus on pACYC184, was kindly provided by Dr. Feng Zhang (Addgene plasmid # 79152; http://n2t.net/addgene: 79152; RRID: Addgene_79152)^14^. In order to generate an efficient vector series carrying a CRISPR-Cas13a system targeting various genes, we conducted vector manipulations. First, a DNA fragment of *cas13a-cas1-cas2* locus was amplified from *L. shahii* strain JCM16776 (ref. ^14^), using a primer set of LsCas13a clo-f and LsCas13a clo-r (Supplementary Table 3), and another DNA fragment from the pC003 vector was amplified, using primers pC003 PCR-r and pC003 PCR-f. The two DNA fragments were then ligated with In-Fusion HD Cloning Kit (Takara Bio Inc., Japan) to generate pKLC5, which resulted in the replacement of *cas13a-cas1-cas2* locus of pC003 with that of JCM16776. Next, the *cas1-cas2* locus was removed from the pKLC5, because Cas1/2 is not necessary for bactericidal activity of CRISPR-Cas13a. This was achieved by performing PCR on the pKLC5 using a primer set of Cas1 Cas2 del SacI-f and Cas1 Cas2 del SacI-r, digesting the PCR fragment with SacI (Takara Bio Inc., Japan) and ligating again with Ligation high ver. 2 (Toyobo Co., Ltd, Japan) to generate pKLC5 ΔCas1/2. Following this, in order to enable the pKLC5 ΔCas1/2 to be packaged into M13 phage capsid, f1 origin together with KanR were inserted as follows: the f1 origin and the KanR were amplified from pRC319, a gift from Timothy Lu (Addgene plasmid # 61272; http://n2t.net/addgene: 61272; RRID: Addgene_61272)^9^, using a primer set of InF13 pRC319-f and InF13 pRC319-r, and another primer set of InF13 PCR SalI-f and InF13 PCR SmaI-r was used to amplify vector part from the pKLC5 ΔCas1/2 vector. The resulting two DNA fragments were then ligated with In-Fusion HD Cloning Kit to generate pKLC21 that carries complete CRISPR-Cas13a system, except for leaving the cloning site empty for insertion of any appropriate spacer. The pKLC21 carries f1 origin of M13 phage thus is able to interact with M13 phage. Finally, a CRISPR-Cas13a system targeting carbapenem-resistant gene *bla*_IMP-1_ was constructed on the pKLC21 vector as follows: briefly, 33 mer oligo DNAs (28 mer of spacer and 5 mer for inserting *Bsa*I cut site) of blaIMP-1_104-s and blaIMP-1_104-as, corresponding to nucleotide positions of *bla*_IMP-1_ from no. 104 to no. 132, were synthesized and annealed in annealing buffer (10 mM Tris-HCl (pH 8.0), 50 mM NaCl, and 1 mM EDTA). After that, pKLC21 was treated with restriction enzyme *Bsa*I-HF, gel purified and subsequently ligated with the annealed oligo DNA using Ligation high ver. 2 to obtain pKLC21_*bla*_IMP-1__104. Likewise, a series of pKLC21 vectors targeting other carbapenem-resistant genes (*bla*_NDM-1_, *bla*_KPC-2_, *bla*_OXA-48_, and *bla*_*VIM-2*_), colistin-resistant genes (*mcr*-1 and *mcr*-2), and *mecA*, *rpsE*, and *ermC* of *S. aureus* were produced. The *bla*_IMP-1_-targeting pKLC21 vector library covering the whole sequences of *bla*_IMP-1_ was also constructed in the same manner, whereby 121 different oligo DNAs pairs covering the whole *bla*_IMP-1_ were inserted into the *Bsa*I site of pKLC21. The sequences of all oligo DNAs used were listed in the Supplementary Table 3. Apart from pKLC21 vector series, *S. aureus*–*E. coli* shuttle vector carrying CRISPR-Cas13a (pKLC3.0) was also constructed. For the assembly of pKLC3.0, three DNA segments, KanR (*aphA-3*) and ori of *E. coli*, *cat* and p15A ori of *S. aureus*, and *cas13a* were amplified from pHel3-FLAG (ref. ^31^), pDB114 (kindly provided by Dr. Luciano Marraffini)^10^ and pKLC21, respectively, using the following primer sets (Supplementary Table 3): InF 3.0 KanR-f and InF 3.0 KanR-r; InF 3.0 SArep_CAT-f and InF 3.0 SArep_CAT-r; and InF 3.0 p15A ori_Cas13-f and InF 3.0 p15A ori_Cas13-r. The resulting three fragments were ligated with In-Fusion HD Cloning Kit to generate pKLC3.0. For the PICImid (plasmid carrying the PICI packaging system) construction, the *cos*N and *rppA* genes (ref. ^25^), which are necessary for the packaging of the island EcCICFT073, were cloned. These were amplified with a primer set of InFpi araCosPPi NotI1-f and InFpi araCosPPi NotI1-r. The resulting fragment was digested with NotI (Takara Bio Inc., Japan) and subsequently cloned into the NotI site of pKLC21 to generate PICImid pKLC31. To construct CRISPR-Cas9 expression vector, pYS29 vector^32^ was digested by PstI. The fragment containing CRISPR-Cas9 was ligated with a PCR fragment of pKLC21 that was amplified using a primer pair InF54 pKLC21 PCR-f and InF54 pKLC21 PCR-r to generate pKLC54. A spacer sequence against *bla*_IMP-1_ was inserted into the BsaI site of pKLC54 vector to obtain pKLC54_*bla*_IMP-1__560 using Ligation high ver. 2. Oligo DNAs used for the spacer construction are listed in Supplementary Table 3.

### *S. aureus*–*E. coli* CRISPR-Cas13a_*mecA* shuttle vector

We constructed the vector pKLC-SP_*mecA* that carries CRISPR-Cas13a_*mecA* sequences flanked by two homologous recombination regions from *bap* (encoding biofilm associated protein) of staphylococcal SaPIbov2 on the *S. aureus*–*E. coli* vector pIMAY. Two PCR fragments of 5′ and 3′ regions of *bap* on SaPIbov2 were first amplified from *S. aureus* strain RN4220-80α-∆*terS*-SaPIbov2::tetM (refs. ^29,33^). The primers BAPup 7272-F and BAPup 8172-R were used for the PCR amplification of the 5′ region of *bap* gene, while primers BAPdown 12224-F and BAPdown 13089-R were used to amplify the 3′ region. The two fragments with 15-bp flanking sequences homologous to vector ends were then sequentially integrated into a temperature-sensitive plasmid pIMAY, using In-Fusion HD Cloning Kit generating pIMAY-*bap*Up/Down. Meanwhile, we used pKLC21_*mecA*-5 as a template for amplification of CRISPR-Cas13a_*mecA* with spacer targeting *mecA* and a CRISPR-Cas13a system devoid of spacer targeting *mecA* (CRISPR-Cas13a_nontargeting). The PCR amplification was carried out with primer pairs of LsC2c2 mecA5-F and LsC2c2 mecA5-R to obtain CRISPR-Cas13a_*mecA*, and LsC2c2 mecA5-F and LsC2c2 188-R to obtain CRISPR-Cas13a_nontargeting (control). Finally, we individually inserted the PCR products between the 5′ and 3′ region of *bap* on pIMAY-*bap*Up/Down with In-Fusion HD Cloning Kit to generate pKLC-SP_*mecA* and pKLC-SP_null (control).

### Construction of target gene expression vectors

We constructed two plasmid systems for expression of target genes of the CRISPR-Cas13a systems: a pSP72 aTc-inducible vector in which the cloning site for target gene expression was under the control of aTc; and pKLC26 that does not contain f1 origin of replication from M13 phage. For the construction of pSP72, aTc-regulatory element was first amplified from pC008 vector using TetReg SalI-f and TetReg BamHI-r primers, and the amplicon was digested with restriction enzymes SalI and BamHI. After digestion, the fragment was inserted into the SalI–BamHI site of pSP72 vector (Promega Corporation, US) carrying pBR322 origin. The resulting plasmid was termed as pSP72 aTc-inducible vector because the cloning site (BamHI/EcoRI) for expression of target gene is regulated by aTc-inducible promoter.

The pC008 (pBR322 with Tet-inducible RFP) was a gift from Feng Zhang (Addgene plasmid # 79157; http://n2t.net/addgene: 79157; RRID: Addgene_79157)^14^. A subsequent set of vectors expressing target genes for CRISPR-Cas13a was produced by cloning AMR genes (e.g., *bla*_IMP-1_, *bla*_NDM-1_, *bla*_KPC-2_, *bla*_OXA-48_, and *bla*_VIM-2_) on the BamHI/EcoRI site of the generated plasmid. The pKLC26 was constructed by modifying pBAD33 with deletion of f1 origin and ARA-promoter. First, pBAD33 vector was amplified with two primers, pBAD33 PCR XhoI-f and pBAD33 PCR XhoI-r, which were designed to exclude f1 origin. After digestion with restriction enzyme XhoI, the fragment was self-ligated with Ligation high ver. 2 to generate pKLC23. Then, the pKLC23 vector and *bla*_IMP-1_ native promoter sequence (1,716-1,168 bp of GenBank ID: AB733642.1 artificially synthesized by GENEWIZ) were independently amplified with two primer sets, pKLC23 PCR InFusion-f2 and pKLC23 PCR InFusion-r2, and InFusion-f and Intl1pro InFusion-r, respectively. Combination of the resulting two fragments with In-Fusion HD Cloning Kit finally generated pKLC26 vector, which carries *bla*_IMP-1_ native promoter in replacement of ARA-promoter.

To obtain pKLC26_*bla*_IMP-1_ for *bla*_IMP-1_ expression, two PCR amplifications were carried out, generating a fragment of pKLC26 using a primer pair InF18 pKLC26-f and InF18 pKLC26-r, and a fragment of *bla*_IMP-1_ (GenBank ID: S71932) using a primer pair InF18 IMP-1-f and InF18 IMP-1-r. The two DNA fragments were then ligated with In-Fusion HD Cloning Kit to yield pKLC26_*bla*_IMP-1_. By using the same method, a series of vectors for expression of the following AMR genes were generated (Supplementary Table 2): *bla*_NDM-1_ (GenBank ID: FN396876), *bla*_KPC-2_ (GenBank ID: AY034847), *bla*_VIM-2_ (GenBank ID: AF191564), *bla*_OXA-48_ (GenBank ID: AY236073), *mcr*-1 (GenBank ID: KP347127), *mcr*-2 (GenBank ID: LT598652), *hygroR* (the sequence was subcloned from TOPO HygroR that was a gift from Tyler Jacks, Addgene plasmid # 68445), and *rfp* (encoding RFP, GenBank ID: KJ021042).

To yield pKLC26_*bla*_IMP-1__*mcr*-2, two PCR amplifications were carried out, generating a fragment of pKLC26_*bla*_IMP-1_ using a primer pair InF55 pKLC26IMP1-f and InF55 pKLC26IMP1-r, and a fragment of pKLC26_*mcr*-2 using a primer pair InF55 pKLC26mcr2-f and InF55 pKLC26mcr2-r. The two DNA fragments were then ligated with In-Fusion HD Cloning Kit. We confirmed the expressions of cloned genes by susceptibility test against corresponding antibiotics using the disk diffusion test recommended by CLSI. To obtain pKLC42 for *hygroR* expression under the control of RecA promoter, PCR amplifications were carried out, generating a fragment of pKLC26_HygroR using a primer pair InF42 HygroR-f and InF42 HygroR-r. The PCR fragment and synthesized RecA promoter oligo DNA (Supplementary Table 3) were then ligated with In-Fusion HD Cloning Kit.

Construction of the pKLC53 was carried out as follows. The RecA promoter was amplified from the pKLC42 using a primer set of InF53 pKLC42 PCR-r and InF53 pKLC42 PCR-f, and the IMP-1 expression sequence was amplified from pKLC26_blaIMP-1, using primers of InF53 IMP-1 PCR-f and InF53 IMP-1 PCR-r. The two amplicons were then ligated with In-Fusion HD Cloning Kit to generate pKLC53. To obtain pKLC56_*bla*_IMP-1_ for *bla*_IMP-1_ expression under the control of Tet-inducible promoter, two PCR amplifications were carried out, generating a fragment of pKLC26_*bla*_IMP-1_ using a primer pair InF56 pKLC26_IMP-1-f and InF56 pKLC26_IMP-1-r, and a fragment of pSP72 aTc-inducible vector using a primer pair InF56 Tet-ind clo-f and InF56 Tet-ind clo-r. The two DNA fragments were then ligated with In-Fusion HD Cloning Kit. By using the same method, aTc-inducible RFP vector and the control vector were generated (Supplementary Table 2).

### Generation of M13 phage-based EC-CapsidM13Cas13a

First, to prevent M13 phage genome from self-assembly, the f1 origin of replication of the helper phage M13KO7 (New England Biolabs, US) was deleted. This was achieved by performing PCR on M13KO7 plasmid using primer pair of M13KO7 PCR InFusion-f and M13KO7 PCR InFusion-r, and subsequently ligating the resulting fragment with another PCR product carrying p15A origin and Cm-resistant gene, which was amplified from pBAD33 using a primer pair of pBAD33 PCR InFusion-f and pBAD33 PCR InFusion-r. The ligation was performed with In-Fusion HD Cloning Kit and the resulting phagemid was termed pKLC25. Next, CRISPR-Cas13a-loaded *E. coli* M13 phage capsid targeting *bla*_IMP-1_ (M13 phage-based EC-CapsidM13Cas13a_*bla*_IMP-1_) was generated as follows: pKLC25 vector was transformed into *E. coli* MC1061 to synthesize helper phage (phage capsid).

The transformed bacteria were selected on Cm plates. Subsequently, the *E. coli* harboring pKLC25 was transformed with pKLC21 that carries CRISPR-Cas13a and f1 origin of M13 (i.e., pKLC21_*bla*_IMP-1_ vector), and selected on the LB agar containing Cm and Km. The colonies grown on this double antibiotic selection plate were picked and cultured in LB liquid medium containing Cm and Km at 37 °C. *E. coli* cultures at stationary phase were then centrifuged at 8000 × *g* for 20 min and the supernatant was passed through a 0.22 µm filter. Equal volume of PEG buffer (5 mM Tris-HCl (pH 7.5), 10% PEG 6000, 1 M NaCl (58 g/L), and 5 mM MgSO_4_·7H_2_O (1.23 g/L)) was added to the filtrate, mixed well, and left at 4 °C for 24 h. After that, mixtures were centrifuged at 12,000 × *g* for 10 min at 4 °C to pellet the M13 phage-based EC-CapsidM13Cas13a_*bla*_IMP-1_. To improve its purity, repeated centrifugation was carried out. Eventually, SM buffer (50 mM Tris-HCl (pH 7.5), 0.1 M NaCl, 7 mM MgSO_4_·7H_2_O, and 0.01% gelatin) was added and the pellet was resuspended to generate a final solution of the M13 phage-based EC-CapsidM13Cas13a_*bla*_IMP-1_. A series of M13 phage-based EC-CapsidM13Cas13a(s) targeting the genes of *bla*_NDM-1_, *bla*_KPC-2_, *bla*_VIM-2_, *bla*_OXA-48_, *mcr*-1, *mcr*-2, and *rfp* were also prepared by using the same methods.

### Generation of PICI-based EC-CapsidCas13a and EC-CapsidCas9

The *E. coli* JP12507 (ref. ^25^), derived by lysogenizing phage Φ80 into *E. coli* 594 strain, was used for the generation of PICI-based EC-CapsidCas13a(s). To prevent Φ80 from being self-packaged during generation of PICI-based EC-CapsidCas13a, the *cos*N site necessary for phage DNA packaging was deleted to create JP17091. By using JP17091 and a series of PICImid pKLC31s, PICI-based EC-CapsidCas13a(s)::KanR targeting various genes were generated as follows. First, the pKLC31_*bla*_IMP-1_ was transformed into JP17091, and the transformants were cultured on Km-containing LB plates. Then, several colonies were isolated and further cultured at 37 °C with shaking in LB liquid medium containing Km together with adding mitomycin C for induction of prophage excision and L-arabinose for induction of *rpp*A (for phage DNA packaging). The L-arabinose was added to a final concentration of 0.2% that allowed it to reach its final concentration of 2 µg/mL when the bacteria culture reached to OD_600_ of 0.1. The cultures were incubated overnight at 30 °C with shaking at 80 r.p.m. After incubation, the supernatant was harvested and passed through a 0.22 µm filter. The same amount of PEG buffer was then added and the solution was left at 4 °C for at least 1 h after being mixed well. Then, the solution was centrifuged at 12,000 × *g* for 10 min at 4 °C, resulting in precipitation of PICI-based EC-CapsidCas13a::KanR_*bla*_IMP-1_.

To remove residual liquids, the pellet was washed several times and finally resuspended in SM buffer for further use. A series of PICI-based EC-CapsidCas13a::KanR(s) targeting the genes of *bla*_NDM-1_, *bla*_KPC-2_, *bla*_VIM-2_, *bla*_OXA-48_, *mcr*-1, *mcr*-2, and *rfp* were also prepared by using the same methods. The PICI-based EC-CapsidCas13a::HygroR carrying *hygroR* instead of *kanR* was generated by using plasmid pKLC44, in which *kanR* was replaced by *hygorR*. We constructed the pKLC44 by cutting the pKLC31 with SacI and XhoI and combining it with *hygroR* amplified from pKLC26 HygroR with the primer pair of InF44 pKLC42-f and InF44 pKLC42-r, using In-Fusion HD Cloning Kit. We generated PICI-based EC-CapsidCas9 targeting *bla*_NDM-1_ (EC-CapsidCas9_*bla*_NDM-1_), using the JP17091 strain and plasmid pKLC27 (carrying CRISPR-Cas9_*bla*_NDM-1_, *rpp*A and packaging site *cos*N). The pKLC27 was constructed as follows. The CRISPR-Cas9_*bla*_NDM-1_ was amplified from the pCR319 (a gift from Timothy Lu)^9^ using primer a set of InF27 pRC319-f and InF27 pRC319-r, and the packaging site *cos*N and *rppA* were amplified from pBAD18 *cos*N *rppA* using primers of InF27 araCosPPi-f and InF27 araCosPPi-r. The two amplicons were then ligated with In-Fusion HD Cloning Kit to generate pKLC27. The vector for PICI-based EC-CapsidCas9 targeting *bla*_IMP-1_ (EC-CapsidCas9_*bla*_IMP-1_) was generated by replacing CRISPR-Cas13a of pKLC31 with CRISPR-Cas9 using two restriction enzymes, SbfI and BglII.

### Generation of SaPI-based SA-CapsidCas13a_*mecA*

The SaPI-based SA-CapsidCas13a_*mecA* was generated basically using a SaPI packaging system^11,30^. First, the *S. aureus–E.coli* shuttle vector pKLC-SP_*mecA* carrying CRISPR-Cas13a_*mecA* targeting *mecA* of MRSA and the control vector pKLC-SP_null were individually transformed into *S. aureus* strain RN4220-80α-∆*terS*-SaPIbov2::tetM^29,33^ by electroporation, using NEPA21 electroporator (Nepa Gene Co., Ltd., Japan) with the following parameters: poring pulse (voltage: 1800 V, pulse length: 2.5 ms, pulse interval: 50 ms, number of pulse: 1, and polarity: +) and transfer pulse (voltage: 100 V, pulse length: 99 ms, pulse interval: 50 ms, number of pulse: 5, and polarity: ±).

The resulting transformants were then recovered at a temperature that permitted plasmid replication (28 °C) for 1 h and plated on tryptic soy agar (TSA) plates supplemented with 10 µg/mL Cm. The plasmid can be integrated into the chromosome by homologous recombination when the transformants were incubated at 37 °C (nonpermissive temperature) in the presence of Cm. To stimulate rolling cycle replication, single colonies (from 37 °C plate) were then inoculated into tryptic soy broth and incubated at 28 °C without antibiotic selection. Finally, 100 µl of the 10^−5^ dilution of this culture was plated on TSA with 1 µg/mL aTc to select cells free of plasmid. Insertion of both CRISPR-Cas13a systems was validated by PCR. Finally, resulting cells were chemically induced by mitomycin C to generate SA-CapsidCas13a_*mecA* and SA-CapsidCas13a_nontargeting (control) by using the method described elsewhere^11^.

### *E. coli* strain with chromosomally integrated genes

The generation of *E. coli* strain expressing foreign gene on its own chromosome was carried out by using ARA-inducible Red recombination system. First, we transformed *E. coli* strains NEB5-alpha F′*I*^q^ (New England Biolabs, US) and MC1061 with plasmid pKD46 that carries ARA-inducible Red recombination system^34^. Next, the desired genes, e.g., *bla*_IMP-1_, were knocked-in into the above strains following the methods described by Tomoya Baba et al.^35^. A DNA fragment containing *bla*_IMP-1_ and Cm resistance gene (*cat*) was amplified from the pKLC26_*bla*_IMP-1_ with a primer pair of K12 genome-in pKLC26-f and K12 genome-in pKLC26 Cm-r, and the PCR product was electroporated into the above *E. coli* cell using ELEPO 21 with the following parameters: poring pulse (voltage: 2000 V, pulse length: 2.5 ms, pulse interval: 50 ms, number of pulses: 1, and polarity: +) and transfer pulse (voltage: 150  V, pulse length: 50 ms, pulse interval: 50 ms, number of pulses: 5, and polarity: ±). After electroporation, 1 mL of SOC was added and the mixture was cultured at 37 °C for 1 h with agitation, before spreading on LB plate containing Cm and further incubated at 37 °C until colonies were observed. The sequence of the resulting insertion was confirmed by Sanger sequencing.

### Bacterial cell growth inhibitory test on plate

The pKLC21_*bla*_IMP-1__563 (Cas13a and crRNA_*bla*_IMP-1__563 expression plasmid) and the pKLC54_*bla*_IMP-1__560 (Cas9 and crRNA_*bla*_IMP-1__560 expression plasmid) targeting *bla*_IMP-1_ were constructed as mentioned above. The CRISPR-Cas expression plasmid was transformed into *E. coli* STBL3(pKLC45) (as a control) and STBL3(pKLC53) (*bla*_IMP-1_ expression plasmid). Each transformant was then plated onto LB plates containing Km, and incubated at 30 °C for 16 h. After that, colonies on the plates were collected and serially diluted with 0.8 % NaCl, and a colony count for the number of surviving cells was carried out.

### Bacterial cell growth inhibitory test in culture medium

The pKLC21_*bla*_IMP-1__563, the pKLC21_*bla*_IMP-1__RFP and the pKLC21_*bla*_IMP-1__nontargeting were transformed into *E. coli* STBL3(pKLC56) (as a control), STBL3(pKLC56_*bla*_IMP-1_) (aTc-inducible *bla*_IMP-1_ expression plasmid), and STBL3(pKLC56_RFP) (aTc-inducible RFP expression plasmid). Each transformant was then plated onto LB plates containing Km and Cm, and incubated at 37 °C for 16 h. The colonies on the plates were transferred to an LB culture medium containing Km and Cm, and incubated at 37 °C for 10 h. After confirming that the bacteria had grown well, each culture was diluted 100-fold with LB medium, and incubated at 30 °C for 1.5 h with vigorous shaking (400 r.p.m.). Then, 1 µg/mL aTc was added to induce the expression of *bla*_IMP-1_ and *rfp*, and incubated at 30 °C for 1 h with vigorous shaking. After that, antibiotics (Km and Cm, and Amp) were added into the culture, and incubated at 30 °C for 23 h with vigorous shaking. OD_650_ of each well was monitored every hour.

### Cell viability test after introducing CRISPR-Cas13a

The pKLC21_*bla*_IMP-1__563 was transformed into *E. coli* STBL3(pKLC56_*bla*_IMP-1_) and the bacteria was plated onto LB plates containing Km and Cm, and incubated at 37 °C for 16 h. The colonies on the plates were transferred to an LB culture medium containing Km and Cm, and incubated at 37 °C for 10 h. The bacterial culture was diluted 100-fold with LB medium, then 1 µg/mL of aTc and the antibiotics Km and Cm were added, followed by incubation at 30 °C for 8 h. The bacteria culture was vigorously shaken for 10 s every 1 h just prior to measuring OD. The bacteria were collected at the time point of 0 h, 1 h, 2 h, 4 h, and 8 h after the treatment, and serially diluted with 0.8% NaCl. Diluted bacteria were spotted on the LB plate without antibiotics and the number of surviving cells were counted.

### Sequence-specific bacterial killing by EC-CapsidCas13a

The logarithmic phase cultures of three *E. coli* strains with overexpression of *bla*_IMP-1_, *mcr-2* in plasmid and carrying control plasmid, NEB5-alpha F′*I*^q^ (pKLC26_*bla*_IMP-1_), and NEB5-alpha F′*I*^q^ (pKLC26_*mcr-2*) and NEB5-alpha F′*I*^q^ (pKLC26), in LB liquid medium were adjusted to an OD_600_ of 0.1, and diluted ten times with fresh LB medium. We transferred 300 μl of each dilution into one tube, mixed well, then divided them into four tubes up to 100 μl for each tube. Three tubes were treated with M13-based EC-CapsidCas13a_*bla*_IMP-1_, EC-CapsidCas13a_*mcr-2*, and EC-CapsidCas13a_nontargeting (control), respectively, while the fourth tube was regarded as non-treated control. EC-CapsidCa13a treatments were carried out by individually adding MOI 100 of the above three CapsidCas13a(s) to their corresponding tubes, mixing well, then culturing at 37 °C with gentle shaking for 6 h. After incubation, serial dilutions were made with 0.8% NaCl and a colony count for the number of survived cells was carried out. The number of colonies that formed on the agar plates containing Amp, colistin, and drug free, respectively, were counted, and cell ratios were calculated. The sequence-specific killing activity of SA-CapsidCas13a_*mecA* was determined by using Tet agar plate, since SA-CapsidCas13a(s) generated were carrying Tet resistance determinant delivered from SaPIbov2 during the packaging processes.

### Measurement of phage titers

The M13 phage/PICI-based EC-CapsidCas13a(s) were serially diluted with SM buffer ranging from 10^−4^ to 10^−7^. In the meantime, overnight culture of *E. coli* strain NEB5-alpha F′*I*^q^ or MC1061 diluted 1:100 with LB broth was incubated with agitation at 37 °C until an OD_600_ of ~0.1 was obtained. Then, 10 µl of each dilution of M13 phage/PICI-based EC-CapsidCas13a(s) was added to 100 µl of bacterial suspension and the mixture was incubated at 37 °C for 30 min. Subsequently, all of the culture solution was plated on LB plates containing Cm or Km, and the plates were incubated overnight at 37 °C. The colonies grown on the Km plate but not on the Cm were counted to calculate the TFU/mL.

### Detection of bacterial genes with CapsidCas13a(s)

The *E. coli* strains to be determined were grown to an OD_600_ of ~0.5. Then, 100 µl of the culture were mixed with 3 mL molten soft agar (LB solution with 0.5% agarose) prewarmed at 50 °C and poured onto an LB plate containing Km or Hygro. The plates were solidified at room temperature. Meanwhile, M13 phage/PICI-based EC-CapsidCas13a with known titers was adjusted to 10^5^ TFU/mL and its tenfold serial dilutions were prepared. Finally, 2 µL of each dilution of the M13 phage/PICI-based EC-CapsidCas13a were spotted onto the solidified soft agar and the plates were incubated at 37 °C. The result was interpreted as positive if bactericidal plaque formed on the plate.

### pKLC21_*bla*_IMP-1_ library sequencing

The pKLC21_*bla*_IMP-1_ (CRISPR-Cas13a_*bla*_IMP-1_ expression plasmid) library targeting the whole region of *bla*_IMP-1_ was constructed as aforementioned. Equal amounts of each of the 121 pKLC21_*bla*_IMP-1_ carrying spacers against different position of *bla*_IMP-1_ and pKLC21_nontargeting (as a control) were mixed and transformed into *E. coli* MC1061(pKLC26) and MC1061(pKLC26_*bla*_IMP-1_). Each transformant was then plated onto 20 LB plates containing Cm and Km, and incubated at 37 °C for 16 h. Next, >10,000 colonies for each transformant were harvested by using LB medium, and plasmids were extracted using QIAGEN Plasmid Midi Kit (QIAGEN). Pair-end sequencing libraries were constructed from the plasmids using Nextera XT Library Prep Kit (Illumina). Sequencing was performed using Illumina MiSeq platform (2 × 301 bp) with MiSeq reagent kit version 3 (Illumina).

### *G. mellonella* survival assay

The M-sized *G. mellonella* larvae purchased from Ikiesa factory (Osaka, Japan) were used for the survival assay to assess the effect of PICI-based EC-CapsidCas13a on treatment of *E. coli* infections. Upon receipt, the larvae were acclimated to the laboratory environment by leaving them in a dark room for 24 h before starting the assay. Larvae with weak movement, dark color, unusual shape, and sizes that differed distinctly from other larvae were excluded from the experiment. A Hamilton syringe (701LT, Hamilton) and a KF 731 needle (Hamilton) were used in this experiment. First, overnight culture of carbapenem-resistant *E. coli* R10-61 carrying the carbapenem resistance gene *bla*_IMP-1_ was diluted at 1:1000 with fresh LB medium and further incubated at 37 °C with agitation to reach an OD_600_ of ~0.5. The bacteria were then washed twice with PBS and adjusted to a density of ~1 × 10^7^ CFU/mL in PBS. Thirty cream-colored larvae for each group were selected and 5 µl of bacterial suspension was directly injected into the left proleg. One hour later, 5 µl of PBS containing MOI 100 of PICI-based EC-CapsidCas13a_*bla*_IMP-1_, EC-CapsidCas13a_nontargeting, or PBS only, were injected into the same site where bacteria solution had been injected. The larvae were transferred to a 37 °C incubator, and the survival was monitored for up to 3 days. Larvae that did not respond to stimulation with needles or whose bodies deformed were counted as dead. Kaplan–Meier survival curves were then generated from data of three independent experiments and analyzed with log-rank test using software EZR (http://www.jichi.ac.jp/saitama-sct/SaitamaHP.files/statmedEN.html).

### Reporting summary

Further information on research design is available in the Nature Research Reporting Summary linked to this Article.

## Supplementary information

Supplementary Information

Peer Review File

Reporting Summary

## Data Availability

The data that support the findings in this study are available upon reasonable request from the corresponding author. Data supporting the findings of this study are available within the Article and Supplementary Information. Bacteria list, plasmid list, primer list, and the screening results are available in the Supplementary Tables. Figures 1c, e, f, 3 and 4, and Supplementary Figs. 1, 2, 6a, e are provided as a Source Data file.

## Source data

Source Data
