# Supplementary material for: Development of CRISPR-Cas13a-based antimicrobials capable of sequence-specific killing of target bacteria
Source: Nat Commun. 2020 Jun 10;11:2934. doi: 10.1038/s41467-020-16731-6 (PMC7287087; doi:10.1038/s41467-020-16731-6)
Supplement: Supplementary file 1 — Supplementary Information [file 41467_2020_16731_MOESM1_ESM.pdf]

Supplementary information for

Development of CRISPR-Cas13a-based antimicrobials capable of sequence-specific killing of target bacteria

Kotaro Kiga et al.

This file contains Supplementary Figure 1-8 and Supplementary Table 1-4.

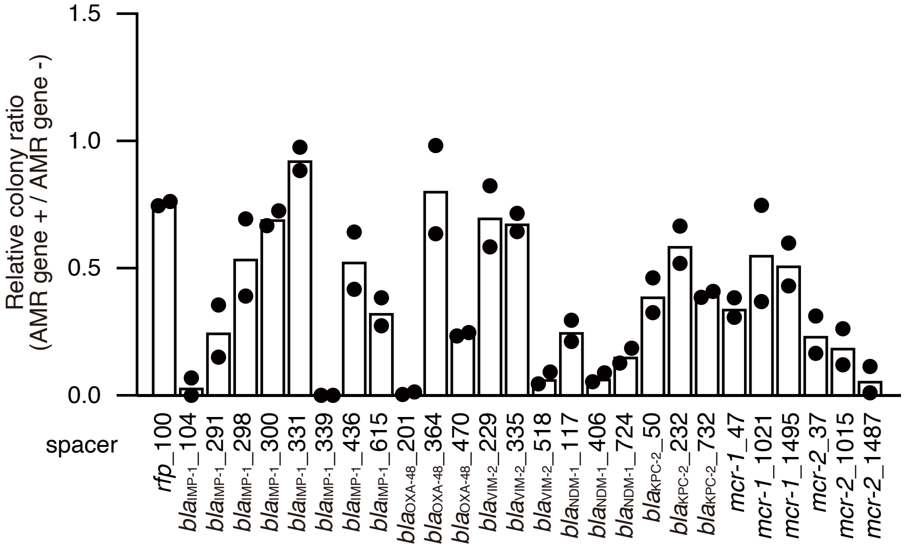

Supplementary Figure 1. Variation in bacterial killing activity of CRISPR-Cas13a system depending on the spacer sequence.

The *E. coli* MC1061 derivatives harboring plasmids expressing different AMR genes (pKLC26_*rfp*, pKLC26_*bla*$_{\mathrm{IMP-1}}$, pKLC26_*bla*$_{\mathrm{OXA-48}}$, pKLC26_*bla*$_{\mathrm{NDM-1}}$, pKLC26_*bla*$_{\mathrm{KPC-2}}$, pKLC26_*mcr-1*, and pKLC26_*mcr-2*) were transformed with CRISPR-Cas13a expression vector pKLC21(s) carrying different spacer sequences. The resultant transformants were plated on agar containing Km and Cm, and incubated at 37°C for 12 hours, then followed by counting the colonies formed on the plates. n = 2.

a

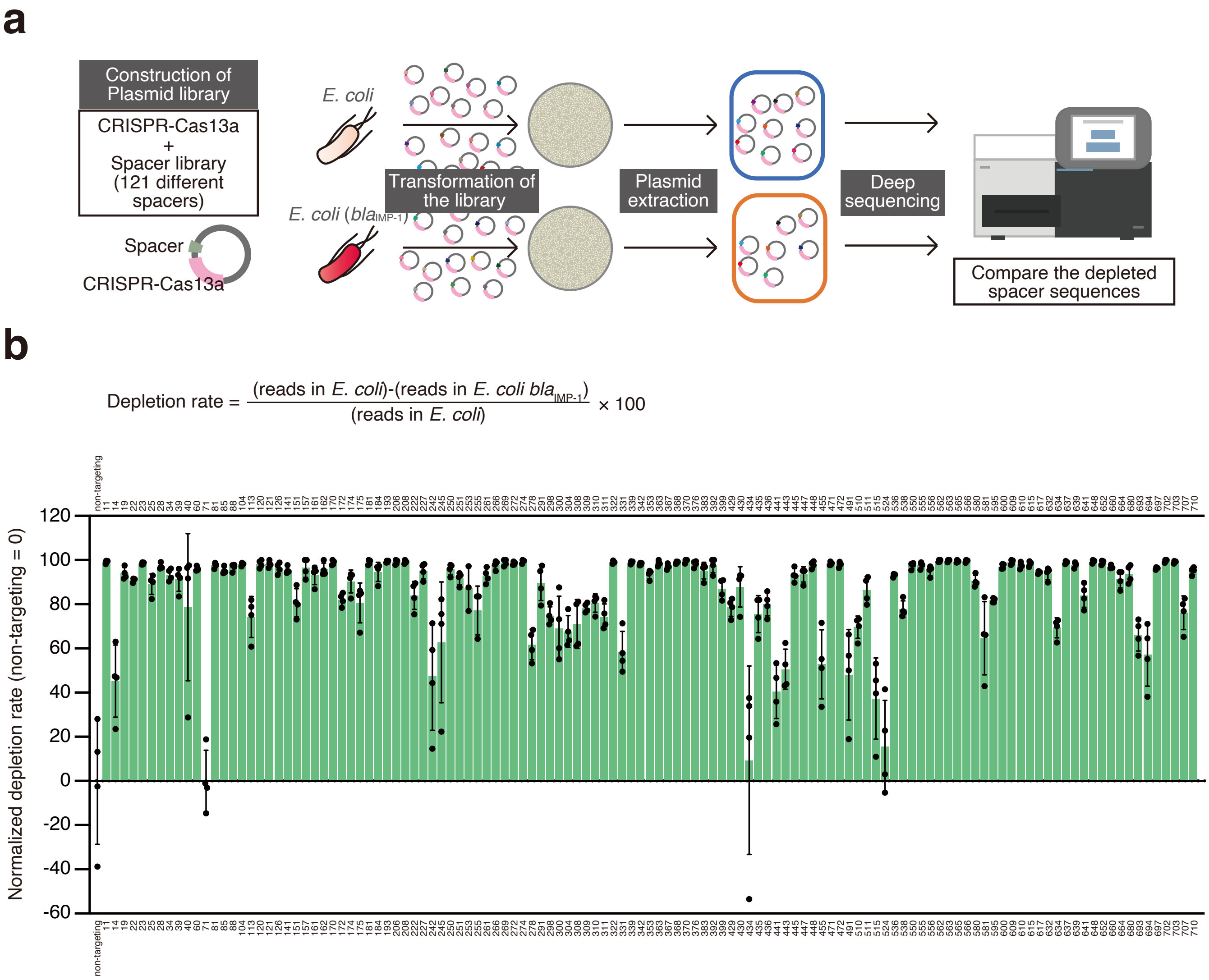

c

No.	Position in IMP-1	Spacer sequence	Depletion rate
1	IMP-1_563	GACTTTGGCCAAGCTTCTATATTTGCGT	99.66
2	IMP-1_702	TTTTGATGGTTTTTTACTTTCGTTTAAC	99.61
3	IMP-1_562	ACTTTGGCCAAGCTTCTATATTTGCGTC	99.60
4	IMP-1_370	TGGCTTGAACCTTACCGTCTTTTTTAAG	99.55
5	IMP-1_566	GCGGACTTTGGCCAAGCTTCTATATTTG	99.55
6	IMP-1_274	TGCTGTCGCTATGAAAATGAGAGGAAAT	99.37
7	IMP-1_565	CGGACTTTGGCCAAGCTTCTATATTTGC	99.36
8	IMP-1_193	TATCTTTAGCCGTAAATGGAGTGTCAAT	99.31
9	IMP-1_648	TTTCAAGAGTGATGCGTCTCCAACTTCA	99.27
10	IMP-1_11	CAAAACAAAAATATAAAGAATACAGATA	99.20
11	IMP-1_703	GTTTTGATGGTTTTTTACTTTCGTTTAA	99.15
12	IMP-1_208	TGACTAACTTTTCAGTATCTTTAGCCGT	99.09
13	IMP-1_609	AACAACCAGTTTTGCCTTACCATATTTG	99.00

Supplementary Figure 2. Evaluation of spacer sequences targeting *bla*$_{IMP-1}$.
a, pKLC21_*bla*$_{IMP-1}$ library with 121 different spacers targeting *bla*$_{IMP-1}$ were constructed and transformed into *E. coli* with and without *bla*$_{IMP-1}$. Plasmids retained in surviving cells were extracted and sequenced to identify the most effective spacer sequence. **b**, Depletion rate of each spacer was calculated by dividing the number of spacer reads of *E. coli* carrying *bla*$_{IMP-1}$ minus that of *E. coli* without *bla*$_{IMP-1}$ by the number of spacer reads of *E. coli* without *bla*$_{IMP-1}$, as shown in the formula. Each bar represents the mean of four biological replicates ± s.d. **c**, The spacer sequences ranked in top 13 with high depletion rate were listed.

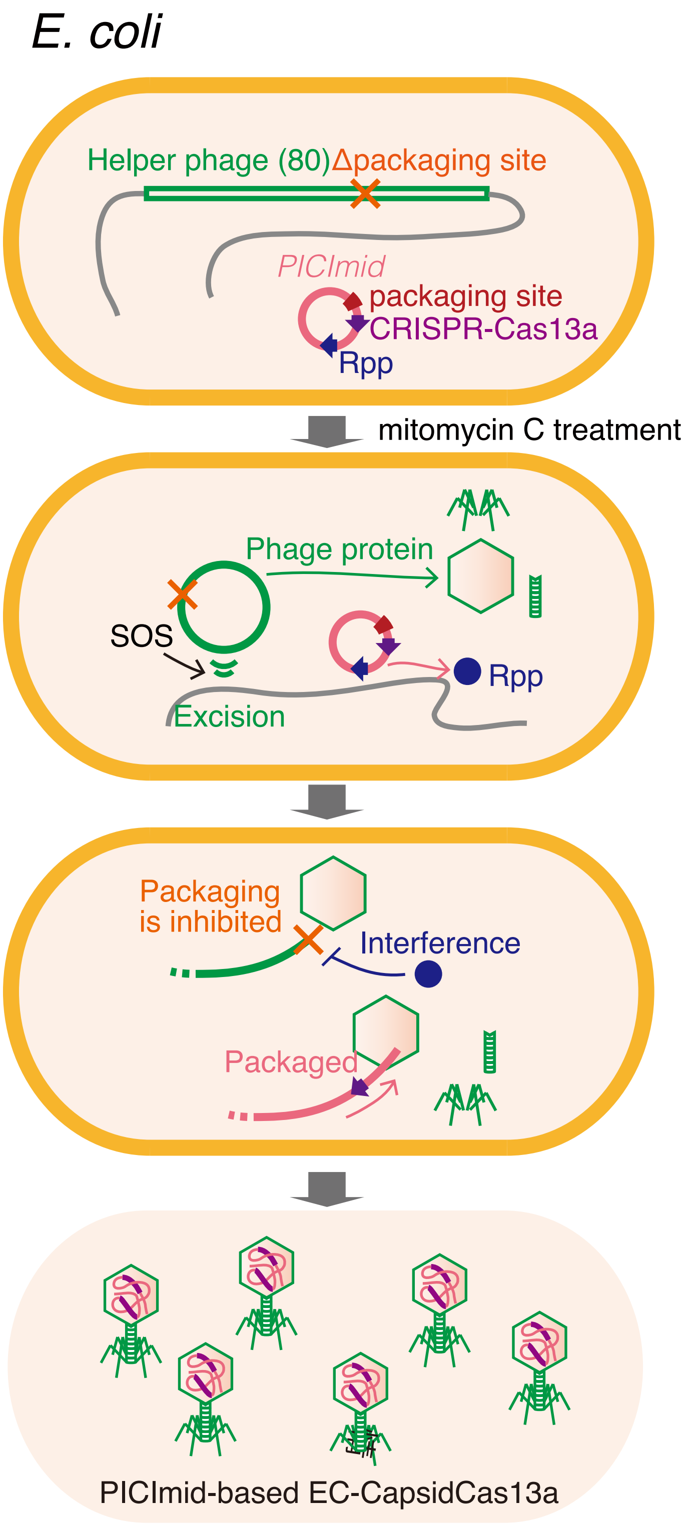

Supplementary Figure 3. Schematic illustration of the construction of PICI-based EC-CapsidCas13a(s) in *E. coli*.
The details about construction procedures are described in the Methods section.

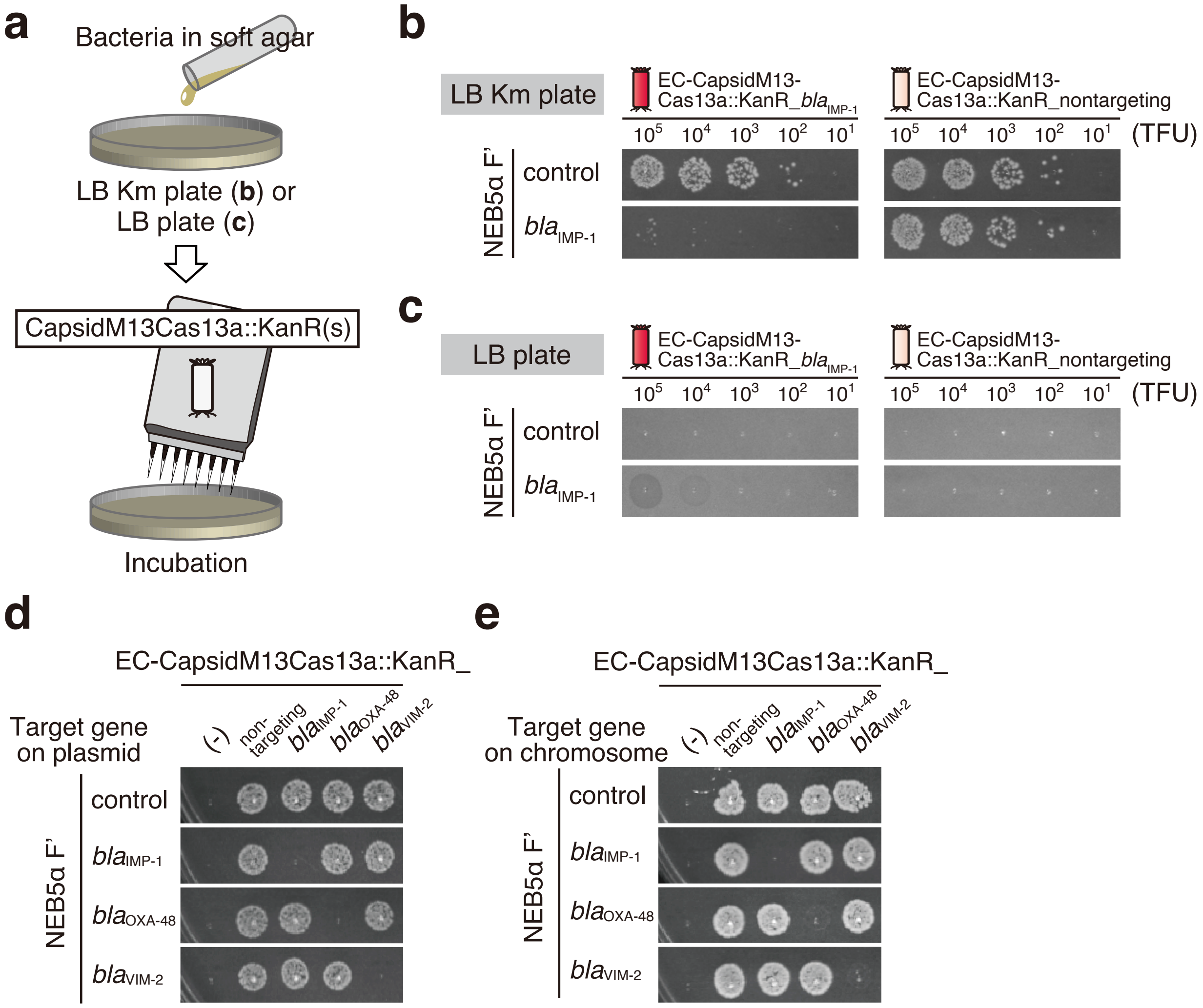

Supplementary Figure 4. M13 phage-based EC-CapsidM13Cas13a as a tool for bacterial gene detection.

a, Schematic illustration demonstrates the bacterial gene detection assay. **b** and **c**, In this assay, bacteria mixed with soft agar were poured onto two types of bottom agar plates, the LB-Km plates (**b**) and the LB plates (**c**), followed by spotting of serially diluted EC-CapsidM13Cas13a(s) carrying Km resistance gene (EC-CapsidM13Cas13a::KanR(s)) onto the surface of the plates. **d** and **e**, The applicability of M13-based EC-CapsidM13Cas13a::KanR(s) for detection of different genotypes of carbapenem resistance genes (*bla*$_{\text{IMP-1}}$, *bla*$_{\text{OXA-48}}$, and *bla*$_{\text{VIM-2}}$) located on both plasmid (**d**) and chromosome (**e**) was tested.

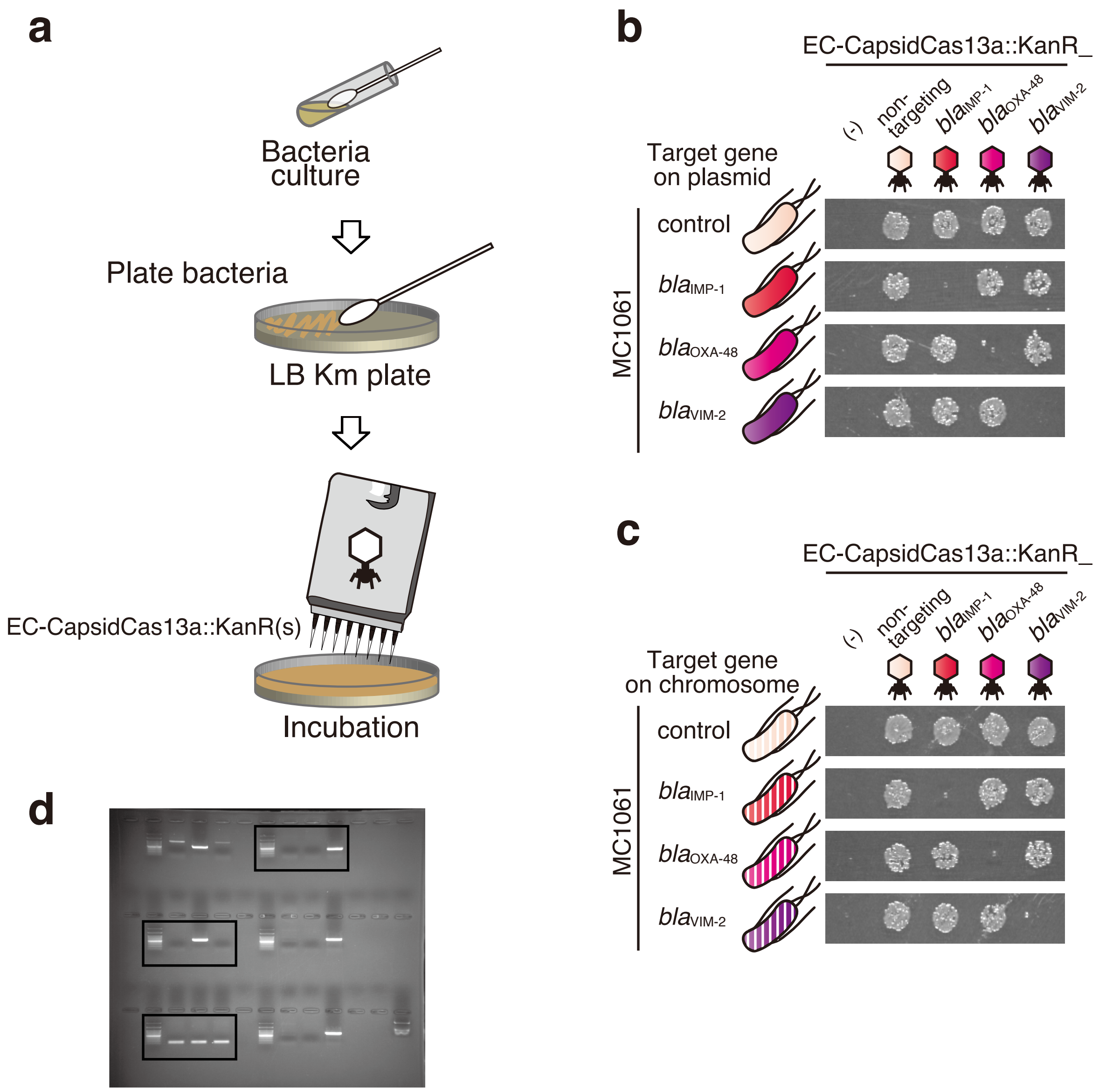

Supplementary Figure 5. Bacterial gene detection using plates prepared by swabbing bacterial cultures directly onto agar plates.
a, Schematic illustration demonstrates the detection of bacterial genes using plate prepared by swabbing bacterial cultures directly onto the agar plate. **b**, Detection results of genes located on plasmid. **c**, Detection results of genes located on bacterial chromosome. These results are correspond to the results of bacterial gene detection using plates prepared by soft agar overlay method (Fig. 5b – 4f). **d**, Full blots picture of Fig. 5j.

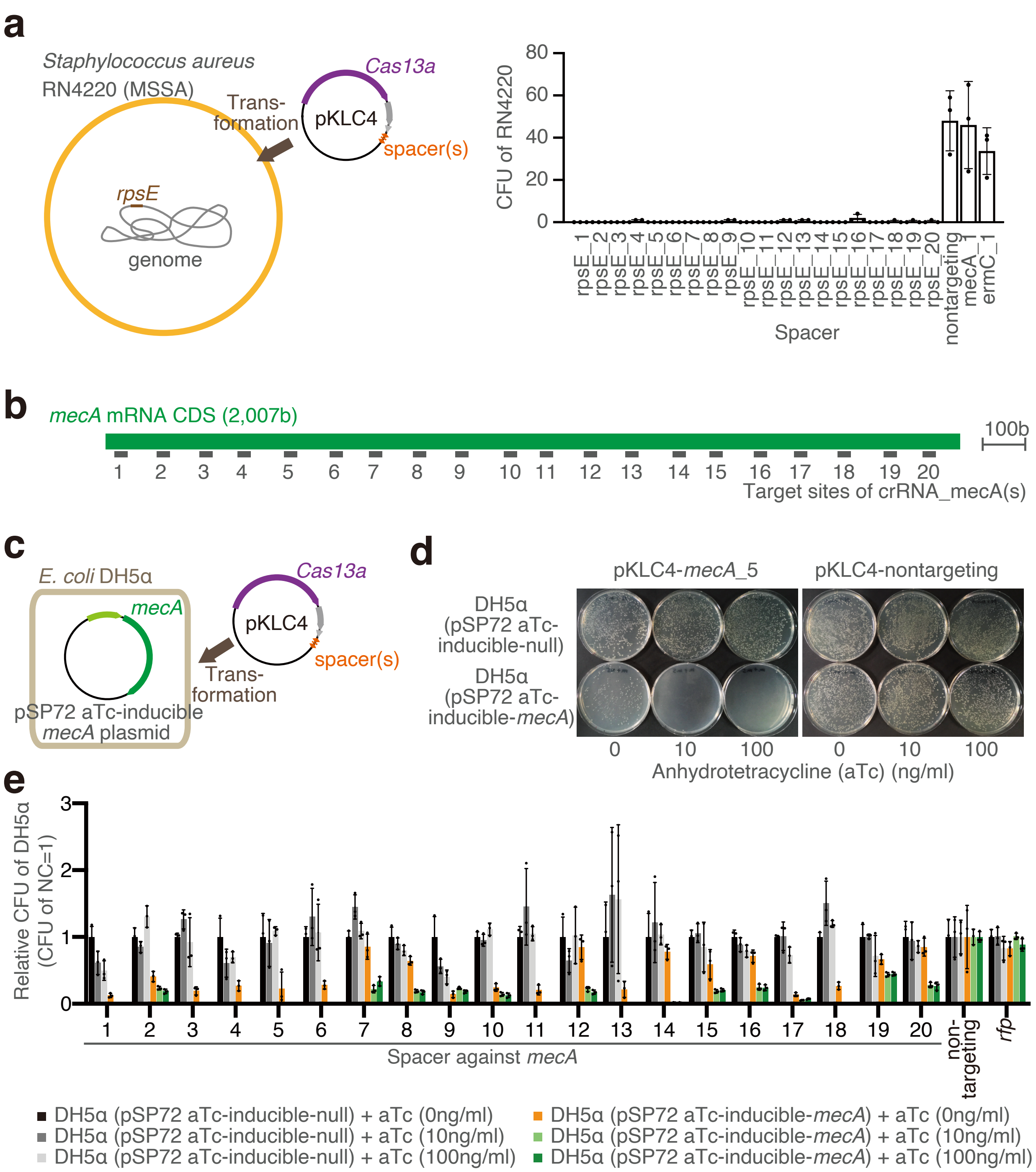

Supplementary Figure 6. Optimization of CRISPR-Cas13a spacer targeting methicillin-resistant gene *mecA* of methicillin-resistant *Staphylococcus aureus* (MRSA).

a, A laboratory strain of *S. aureus* RN4220 with *rpsE* but without *ermC* and *mecA* was transformed with a series of pKLC4(s) carrying CRISPR-Cas13a targeting *rpsE* (20 locations), *ermC* (one location) and *mecA* (one location), respectively. The resulting transformants were cultured on the TSA plate containing Cm and CFU of survived cells were counted. Note that CRISPR-Cas13a showed sequence-specific killing activity against *S. aureus* (right panel). Each bar represents the mean of three biological replicates ± s.d. **b – e**, To optimize spacer of CRISPR-Cas13a (CRISPR-Cas13a_*mecA*) targeting *mecA* of MRSA, pKLC4_*mecA*(s) with 20 different spacers targeting different position of *mecA* (**b**), and without spacer as control (non-targeting control), were constructed and transformed into *E. coli* DH5α carrying or without carrying anhydrotetracycline-inducible *mecA*-expression vector (**c**). The resulting transformants above (c) were evaluated for selecting the best spacer by culturing on the plates under condition of induction of *mecA*-expression. A representative result of the test with the best spacer (No. 5) (**d**), and relative CFU for all 20 spacers normalized with CFU of non-targeting control (**e**) were presented. Each bar represents the mean of three biological replicates ± s.d.

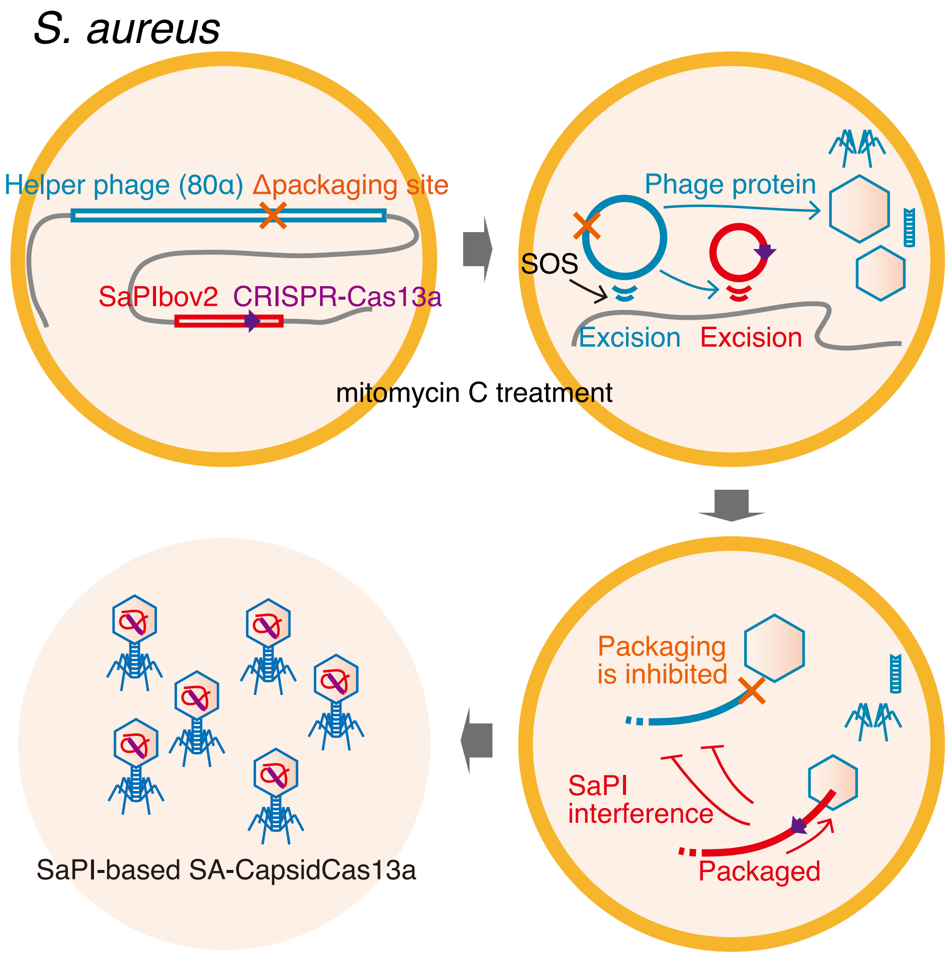

Supplementary Figure 7. Schematic illustration of the SaPI-based SA-CapsidCas13a construction.
The details about construction procedures are described in the Methods section.

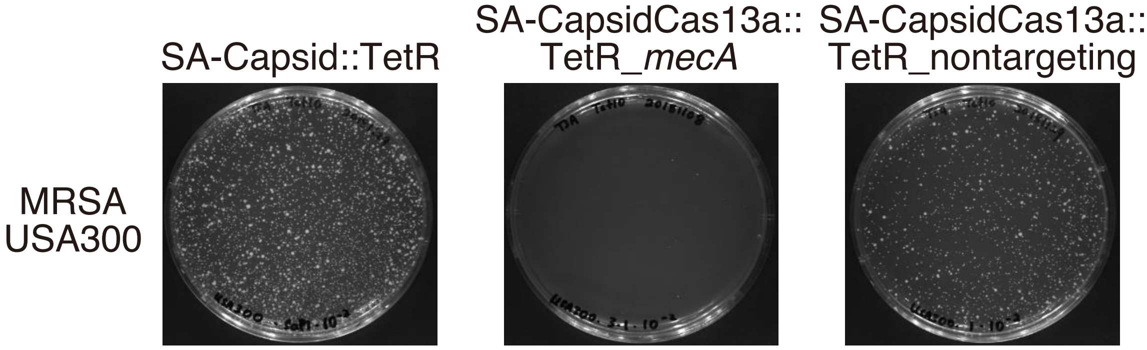

Supplementary Figure 8. Sequence-specific bacterial killing activity of CapsidCas13a against MRSA by targeting *mecA*.
Bacterial lawns of MRSA USA300 carrying *mecA* were treated by SA-Capsid with CRISPR-Cas13a targeting *mecA* (SA-CapsidCas13a::TetR-*mecA*) (middle), non-CRISPR-Cas13a control (SA-Capisid::TetR) (left), and CRISPR-Cas13a carrying non-targeting spacer control (SA-CapsidCas13a::TetR-nontargeting) (right), respectively, and cultured on the TSA plates containing Tet. All the SA-Capsids above carry Tet-resistant gene. Note that the SA-CapsidCas13a that carries spacer targeting *mecA* only killed MRSA.

Table S1. List of Bacterial strains used in this study

Bacterial strain	Description	Origin
STBL3	F-, mcrB , mrr, hsdS20(rB-, mB-), recA13, supE44, ara-14, galK2, lacY1, proA2, rpsL20(StrR), xyl-5, λ-, leu, mtl-1	Thermo Fisher Scientific[a]
MC1061	hsdR, mcrB, araD139, Δ(araABC-leu)7679, ΔlacX74, galU, galK, rpsL, thi	Casadaban et al., 1980[b]
NEB5-alpha F' I^q	F' proA+B+ lacIq Δ(lacZ)M15 zzf::Tn10 (TetR) / fhuA2Δ(argF-lacZ)U169 phoA glnV44 Φ80Δ(lacZ)M15 gyrA96 recA1 relA1 endA1 thi-1 hsdR17	NEB biolabs[c]
STBL3 (pControl)	STBL3 transformed with pKLC53	in this study
STBL3 (p*bla*$_{\text{IMP-1}}$)	STBL3 transformed with pKLC53-*bla*$_{\text{IMP-1}}$	in this study
STBL3 *bla*$_{\text{IMP-1}}$ in chromosome	STBL3; *bla*$_{\text{IMP-1}}$ sequence (from pKLC53) was inserted in the chromosome	in this study
STBL3 control (null) in chromosome	STBL3; pKLC53 was inserted in the chromosome	in this study
STBL3 (pKLC56)	STBL3 transformed with pKLC56	in this study
STBL3 (pKLC56-*rfp*)	STBL3 transformed with pKLC56-*rfp*	in this study
STBL3 (pKLC56-*bla*$_{\text{IMP-1}}$)	STBL3 transformed with pKLC56-*bla*$_{\text{IMP-1}}$	in this study
NEB5α F' (pPBAD-control)	NEB5-alpha F' lq transformed with pKLC23	in this study
NEB5α F' (pPBAD-*bla*$_{\text{IMP-1}}$)	NEB5-alpha F' lq transformed with pKLC23-*bla*$_{\text{IMP-1}}$	in this study
NEB5α F' (pControl)	NEB5-alpha F' lq transformed with pKLC26	in this study
NEB5α F' (p*bla*$_{\text{IMP-1}}$)	NEB5-alpha F' lq transformed with pKLC26-*bla*$_{\text{IMP-1}}$	in this study
NEB5α F' (p*bla*$_{\text{OXA-48}}$)	NEB5-alpha F' lq transformed with pKLC26-*bla*$_{\text{OXA-48}}$	in this study
NEB5α F' (p*bla*$_{\text{VIM-2}}$)	NEB5-alpha F' lq transformed with pKLC26-*bla*$_{\text{VIM-2}}$	in this study
NEB5α F' (p*bla*$_{\text{NDM-1}}$)	NEB5-alpha F' lq transformed with pKLC26-*bla*$_{\text{NDM-1}}$	in this study
NEB5α F' (p*bla*$_{\text{KPC-2}}$)	NEB5-alpha F' lq transformed with pKLC26-*bla*$_{\text{KPC-2}}$	in this study
NEB5α F' (p*mcr-1*)	NEB5-alpha F' lq transformed with pKLC26-*mcr-1*	in this study
NEB5α F' (p*mcr-2*)	NEB5-alpha F' lq transformed with pKLC26-*mcr-2*	in this study
NEB5α F' *bla*$_{\text{NDM-1}}$ in chromosome	MC1061; *bla*$_{\text{NDM-1}}$ sequence was inserted in the chromosome	in this study
R10-61	Carbapenem-resistant E. coli	Hibiki Research Group for Clinical Microbiology
R10-79	Carbapenem-resistant E. coli	Hibiki Research Group for Clinical Microbiology
MC1061 (pControl)	MC1061 transformed with pKLC26	in this study
MC1061 (p*bla*$_{\text{IMP-1}}$)	MC1061 transformed with pKLC26-*bla*$_{\text{IMP-1}}$	in this study
MC1061 (p*bla*$_{\text{OXA-48}}$)	MC1061 transformed with pKLC26-*bla*$_{\text{OXA-48}}$	in this study
MC1061 (p*bla*$_{\text{VIM-2}}$)	MC1061 transformed with pKLC26-*bla*$_{\text{VIM-2}}$	in this study
MC1061 (p*bla*$_{\text{IMP-1}}$_*mcr* -2)	MC1061 transformed with pKLC26-*bla*$_{\text{IMP-1}}$_mcr-2	in this study
MC1061 *bla*$_{\text{IMP-1}}$ in chromosome	MC1061; *bla*$_{\text{IMP-1}}$ sequence was inserted in the chromosome	in this study
MC1061 *bla*$_{\text{OXA-48}}$ in chromosome	MC1061; *bla*$_{\text{OXA-48}}$ sequence was inserted in the chromosome	in this study
MC1061 *bla*$_{\text{VIM-2}}$ in chromosome	MC1061; *bla*$_{\text{VIM-2}}$ sequence was inserted in the chromosome	in this study
MC1061 (p*stx-1*)	MC1061 transformed with pKLC26-*stx-1* (partial)	in this study
MC1061 (p*stx-2*)	MC1061 transformed with pKLC26-*stx-2* (partial)	in this study
RN4220	Laboratory strain of *S. aureus*	Kreiswirth BN et al., 1983[d]
USA300	Methicillin-resistant *S. aureus* (MRSA), FPR3757	Diep BA et al., 2006[e]
USA300 Δ*mecA*	*mecA* deletion mutant of USA300	in this study

a. Thermo Fischer Scientific (https://thermofisher.com/)

b. M. J. Casadaban, S. N. Cohen. Analysis of gene control signals by DNA fusion and cloning in Escherichia coli. J Mol Biol. (1980)

c. New England Biolabs (https://international.neb.com/)

d. B. N. Kreiswirth, S. Löfdahl, M. J. Betley, M. O'Reilly, P. M. Schlievert, M. S. Bergdoll, R. P. Novick, The toxic shock syndrome exotoxin structural gene is not detectably transmitted by a prophage. Nature (1983), doi:10.1038/305709a0

e. B.A. Diep, S.R. Gill, R. F. Chang, T. H. Phan, J. H. Chen, M. G. Davidson, F. Lin, J. Lin, H. A. Carleton, E. F. Mongodin, G. F. Sensabaugh, F. Perdreau-Remington, Complete genome sequence of USA300, an epidemic clone of community-acquired meticillin-resistant Staphylococcus aureus. Lancet (2006) doi:10.1016/S0140-6736(06)68231-7

Table S2. List of Vectors used in this study

Vectetor	Purpose	Discription	Origin
pSP72	Construction of plasmid	Cloning vector, multiple cloning site, β-lactamase, SP6 promoter, T7 promoter	Promega[a]
pC003		Cas13 expression vector, Cas1, Cas2, p15A ori, TcR, CAT	From Dr. Feng Zhang[b]
pDB114		Cas9 expression vector in S. aureus, CAT, rep from pC194	From Dr. Luciano A. Marraffini[c]
pRC319		Cas9 expression vector, pBR322 ori, F1 ori, KanR, crRNA for blaNDM-1	From Dr. Timothy Lu[d]
pKLC3.0		Shuttle vector for Cas13a expression in E. coli and S. aureus, p15A ori for E. coli, KanR (E. coli), CAT (S. aureus), rep for S. aureus (pC194)	This study
pKLC4		Shuttle vector for CRISPR-Cas13a expression in E. coli and S. aureus, p15A ori for E. coli, KanR (E. coli), CAT (S. aureus), rep for S. aureus (pC194) in this study	This study
pSP72 aTc-inducible	Tetracycline-inducible target gene expression vector	Expression vector under the control of anhydrotetracycline-inducible promoter, pBR322 ori, beta-lactamase	This study
pSP72 aTc-inducible *mecA*		*mecA* expression vector under the control of anhydrotetracycline-inducible promoter, pBR322 ori, beta-lactamase	This study
pSP72 aTc-inducible *bla* IMP-1		*bla* IMP-1 expression vector under the control of anhydrotetracycline-inducible promoter, pBR322 ori, beta-lactamase	This study
pKLC21	Construction of M13-based CapsidCas13a::KanR	CRISPR-Cas13a expression vector in E. coli, ColE1 ori, KanR, f1 ori	This study
pKLC21_*blaI* MP-1_339		CRISPR-Cas13a (target: *bla* IMP-1_339) expression vector in E. coli, ColE1 ori, KanR, f1 ori	This study
pKLC21_*blaI* MP-1_563		CRISPR-Cas13a (target: *bla* IMP-1_563) expression vector in E. coli, ColE1 ori, KanR, f1 ori	This study
pKLC21_*bla* oxa-48_201		CRISPR-Cas13a (target: *bla* OXA-48_201) expression vector in E. coli, ColE1 ori, KanR, f1 ori	This study
pKLC21_*bla* VIM-2_518		CRISPR-Cas13a (target: *bla* VIM-2_518) expression vector in E. coli, ColE1 ori, KanR, f1 ori	This study
pKLC21_*bla* NDM-1_406		CRISPR-Cas13a (target: *bla* NDM-1_406) expression vector in E. coli, ColE1 ori, KanR, f1 ori	This study
pKLC21_*bla* KPC-2_50		CRISPR-Cas13a (target: *bla* KPC-2_50) expression vector in E. coli, ColE1 ori, KanR, f1 ori	This study
pKLC21_*mcr-1* _47		CRISPR-Cas13a (target: *mcr-1* _50) expression vector in E. coli, ColE1 ori, KanR, f1 ori	This study
pKLC21_*mcr-2* _1487		CRISPR-Cas13a (target: *mcr-2* _1487) expression vector in E. coli, ColE1 ori, KanR, f1 ori	This study
pKLC23	Arabinose-inducible target gene expression vector	Antibiotics resistance gene expression vector, p15A ori, PBAD ptomoter, CAT	This study
pKLC25	Construction of M13 phage	M13 helper phage vector, p15A ori, CAT, f1 ori is deleted, E. coli transformed with this vector grow slowly	This study
pKLC26	Constitutive target gene expression vector	Target gene expression vector, p15A ori, intI1 promoter (native promoter of *bla* IMP-1), CAT	This study
pKLC26_*blaI* MP-1		*bla* IMP-1 expression vector, p15A ori, intI1 promoter (native promoter of *bla* IMP-1), CAT	This study
pKLC26_*bla* oxa-48		*bla* OXA-48 expression vector, p15A ori, intI1 promoter (native promoter of *bla* IMP-1), CAT	This study
pKLC26_*bla* VIM-2		*bla* VIM-2 expression vector, p15A ori, intI1 promoter (native promoter of *bla* IMP-1), CAT	This study
pKLC26_*bla* NDM-1		*bla* NDM-1 expression vector, p15A ori, intI1 promoter (native promoter of *bla* IMP-1), CAT	This study
pKLC26_*bla* KPC-2		*bla* KPC-2 expression vector, p15A ori, intI1 promoter (native promoter of *bla* IMP-1), CAT	This study
pKLC26_*mcr-1*		*mcr-1* expression vector, p15A ori, intI1 promoter (native promoter of *bla* IMP-1), CAT	This study
pKLC26_*mcr-2*		*mcr-2* expression vector, p15A ori, intI1 promoter (native promoter of *bla* IMP-1), CAT	This study
pKLC26_*mcr-3*		*mcr-3* expression vector, p15A ori, intI1 promoter (native promoter of *bla* IMP-1), CAT	This study
pKLC26_*mcr-4*		*mcr-4* expression vector, p15A ori, intI1 promoter (native promoter of *bla* IMP-1), CAT	This study
pKLC26_*mcr-5*		*mcr-5* expression vector, p15A ori, intI1 promoter (native promoter of *bla* IMP-1), CAT	This study

pKLC26_*blaI* MP-1_*mcr-2*		*bla* IMP-1 and *mcr-2* expression vector, p15A ori, intl1 promoter (native promoter of *bla* IMP-1), CAT	This study
pKLC26_*stx-1* (partial)		*stx-1* (partial) expression vector, p15A ori, intl1 promoter (native promoter of *bla* IMP-1), CAT	This study
pKLC26_*stx-2* (partial)		*stx-2(partial)* expression vector, p15A ori, intl1 promoter (native promoter of *bla* IMP-1), CAT	This study
pKLC31	Construction of PICI-based CapsidCas13a::KanR	PICI-construction vector, CRISPR-Cas13a, KanR, pBR322 ori, aTc-inducible c1501/c1502/c1503	This study
pKLC31_*blaI* MP-1_339		PICI-construction vector, CRISPR-Cas13a (target: *bla* IMP-1_339), KanR, pBR322 ori, aTc-inducible c1501/c1502/c1503	This study
pKLC31_*blaI* MP-1_563		PICI-construction vector, CRISPR-Cas13a (target: *bla* IMP-1_563), KanR, pBR322 ori, aTc-inducible c1501/c1502/c1503	This study
pKLC31_*bla* oxa-48_201		PICI-construction vector, CRISPR-Cas13a (target: *bla* OXA-48_201), KanR, pBR322 ori, aTc-inducible c1501/c1502/c1503	This study
pKLC31_*bla* VIM-2_518		PICI-construction vector, CRISPR-Cas13a (target: *bla* VIM-2_518), KanR, pBR322 ori, aTc-inducible c1501/c1502/c1503	This study
pKLC31_*bla* NDM-1_406		PICI-construction vector, CRISPR-Cas13a (target: *bla* NDM-1_406), KanR, pBR322 ori, aTc-inducible c1501/c1502/c1503	This study
pKLC31_*bla* KPC-2_50		PICI-construction vector, CRISPR-Cas13a (target: *bla* KPC-2_50), KanR, pBR322 ori, aTc-inducible c1501/c1502/c1503	This study
pKLC31_*mcr-1* _47		PICI-construction vector, CRISPR-Cas13a (target: *mcr-1* _47), KanR, pBR322 ori, aTc-inducible c1501/c1502/c1503	This study
pKLC31_*mcr-2* _1487		PICI-construction vector, CRISPR-Cas13a (target: *mcr-2* _1487), KanR, pBR322 ori, aTc-inducible c1501/c1502/c1503	This study
pKLC31_stx-*1* _640		PICI-construction vector, CRISPR-Cas13a (target: *mcr-1* _47), KanR, pBR322 ori, aTc-inducible c1501/c1502/c1503	This study
pKLC31_stx-*2* _640		PICI-construction vector, CRISPR-Cas13a (target: *mcr-2* _1487), KanR, pBR322 ori, aTc-inducible c1501/c1502/c1503	This study
pKLC42	Construction of PICI-based CapsidCas13a::HygroR	HygroR expression vector, RecA promoter, p15A ori, CAT	This study
pKLC44		PICI-construction vector, CRISPR-Cas13a, HygroR, pBR322 ori, aTc-inducible c1501/c1502/c1503	This study
pKLC44_*bla* IMP-1_563		PICI-construction vector, CRISPR-Cas13a (target: *bla* IMP-1_563), HygroR, pBR322 ori, aTc-inducible c1501/c1502/c1503	This study
pKLC44_*bla* NDM-1_406		PICI-construction vector, CRISPR-Cas13a (target: *bla* NDM-1_406), HygroR, pBR322 ori, aTc-inducible c1501/c1502/c1503	This study
pKLC53	Constitutive target gene expression vector	RecA promoter, p15A ori, CAT	This study
pKLC53_blaIMP-1		*bla* IMP-1 promoter, p15A ori, CAT	This study
pKLC54	CRISPR-Cas9 expression vector construction	CRISPR-Cas13a (nontargeting) expression vector in E. coli, ColE1 ori, KanR, f1 ori	This study
pKLC54_blaIMP-1_560		CRISPR-Cas9 (target: blaIMP-1_560) expression vector in E. coli, ColE1 ori, KanR, f1 ori	This study
pYS29		CRISPR-Cas9 expression vector, ori (EC), ori (SA), CAT	Sato'o *et al.* 2018[e]
pKLC56	Arabinose-inducible target gene expression vector	Expression vector under the control of anhydrotetracycline-inducible promoter, p15A ori, CAT	This study
pKLC56-*rfp*		RFP expression vector under the control of anhydrotetracycline-inducible promoter, p15A ori, CAT	This study
pKLC56_*bla* IMP-1		blaIMP-1 Expression vector under the control of anhydrotetracycline-inducible promoter, p15A ori, CAT	This study
pC003-*mecA* 5	Construction of *mecA* -targeting Cas13a	pC003 loaded with optimized crRNA targeting *mecA*	This study
pC003-*mecA* 5-ΔCas1/2		pC003-*mecA* 5 with deleted Cas1 and Cas2	This study
pIMAY	Construction of SaPI-based CapsidCas13a::TetR	*E. coli* /staphylococcal temperature-sensitive plasmid, ori for *E. coli* p15A, P*help-cat* , anti-*secY* , temperature-sensitive replicon for Gram-positive bacteria (*repBCAD*)	Monk *et al.* 2012[f]
pIMAY-BAPup/down		pIMAY carrying 5' and 3' flanking regions of *bap* gene	This study
pIMAY-LshCas13a-SP*mecA*		pIMAY-BAPup/down with LshCas13a and crRNA targeting *mecA* inserted in between 5' and 3' flanking regions of *bap* gene	This study
pIMAY-LshCas13a-null		pIMAY-BAPup/down with only LshCas13a inserted in between 5' ar	This study
pIMAY-KO*mecA*		pIMAY carrying 5' and 3' flanking regions of *mecA* gene	This study

a. Thermo Fisher Scientific (Promega) (https://www.fishersci.ca/shop/products/promega-psp72-vector/prp2191)

b. O. O. Abudayyeh, J. S. Gootenberg, S. Konermann, J. Joung, I. M. Slaymaker, D. B. T. Cox, S. Shmakov, K. S. Makarova, E. Semenova, L. Minakhin, K. Severinov, A. Regev, E. S. Lander, E. v. Koonin, F. Zhang, C2c2 is a single-component programmable RNA-guided RNA-targeting CRISPR effector. Science (2016), doi:10.1126/science.aaf5573.

c. D. Bikard, C. W. Euler, W. Jiang, P. M. Nussenzweig, G. W. Goldberg, X. Duportet, V. A. Fischetti, L. A. Marraffini, Exploiting CRISPR-cas nucleases to produce sequence-specific antimicrobials. Nature Biotechnology (2014), doi:10.1038/nbt.3043.

d. R. J. Citorik, M. Mimee, T. K. Lu, Sequence-specific antimicrobials using efficiently delivered RNA-guided nucleases. Nature Biotechnology (2014), doi:10.1038/nbt.3011.

e. Sato'o Y, Hisatsune J, Yu L, Sakuma T, Yamamoto T, Sugai M. Tailor-made gene silencing of Staphylococcus aureus clinical isolates by CRISPR interference.

f. I. R. Monk, I. M. Shah, M. Xu, M. W. Tan, T. J. Foster, Transforming the untransformable: application of direct transformation to manipulate genetically Staphylococcus aureus and Staphylococcus epidermidis. MBio (2012), doi: 10.1128/mBio.00277-11.

Table S3. List of Primers used in this study

Purpose	Primer name	Sequence
Deletion of Cas1/2 from pC003	Cas1Cas2 del SacI-f	ATATGAGCTCATGGGAGAAAAAATTTCACAAAAC
	Cas1Cas2 del SacI-r	ATATGAGCTCTCATTCTTATAACGTATCATTCG
Construction of pKLC3.0	InF3.0 KanR-f	gcattaaagctcgtttaacagcGTTTTTAGTTGAAAGCTAACTTC
	InF3.0 KanR-r	ttccacattttcccaCAGTGAATTGGAGTTCGTC
	InF3.0 SArep_CAT-f	acactccgctagcgcCAAACGAAAATTGGATAAAGTG
	InF3.0 SArep_CAT-r	gctgttaaacgagctttaatgcCGTTTGTTGAACTAATGGGTG
	InF3.0 p15A ori_Cas13-f	TGGGAAAATGTGGAATTTGAAAC
	InF3.0 p15A ori_Cas13-r	GCGCTAGCGGAGTGTATACTG
Construction of pKLC21	InF13 SalI PCR-f	tcttcaccctgtcgatgggaaaatgtggaatttg
	InF13 SmaI PCR-r	caggatcttctgcccaattaggctctagttagcct
	InF13 pRC319-f	gggcagaagatcctgcagg
	InF13 pRC319-r	tcgacagggtgaagacgaaag
Construction of pKLC21 IMP-1_104	pC003 blaIMP-1_104-s	tatccATGTTCATACTTCGTTTGAAGAAGTTAA
	pC003 blaIMP-1_104-as	aaacTTAACTTCTTCAAACGAAGTATGAACATg
Construction of pKLC25	M13KO7 PCR InFusion-f	cctattggttaaaaaatgagctg
	M13KO7 PCR InFusion-r	actatggttgctttgacgag
	pBAD33 PCR InFusion-f	caaagcaaccatagtgtagcaccaggcgtttaagg
	pBAD33 PCR InFusion-r	tttttaaccaataggcatcaccgatggggaagatc
Construction of pKLC23	pBAD33 PCR InFusion-f	tcaCTCGAGcgaatttgctttcgaatttc
	pBAD33 PCR InFusion-r	tcaCTCGAGcaaaagagtttgtagaaacgc
Construction of pKLC26	pKLC23 PCR InFusion-f	GAATTCgatcctctagagtc
	pKLC23 PCR InFusion-r	tgcttcgtccatttgacag
	Intl1pro PCR InFusion-f	caaatggacgaagcaTGACGCACACCGTGGAAAC
	Intl1pro PCR InFusion-r	tagaggatcGAATTCGAGAATGGATTTTGTGATGC
Construction of pKLC54	InF54 pKLC21 PCR-r	tgagcgcattgttactgcagtccttctttcgcctttagatcc
	InF54 pKLC21 PCR-f	gaacatatccatcgctgcagcctaattgggcagaagatcctg
Construction of pKLC53	InF53 pKLC42 PCR-r	ggtgggtacctcctTTGAATTCTG
	InF53 pKLC42 PCR-f	GAATTCgatcctctagagtcgacctg
	InF53 IMP-1 PCR-f	AaggaggtacccaccATGAGCAAGTTATCTGTATTC
	InF53 IMP-1 PCR-r	tagaggatcGAATTCTTAGTTGCTTGGTTTTGATGG
Construction of antibiotics resistant gene expression vector	InF18 pKLC26-f	GAATTCgatcctctagagtc
	InF18 pKLC26-r	GAGAATGGATTTTGTGATGC
	InF18 NDM-1-f	ACAAAATCCATTCTCATGGAATTGCCCAATATTATG
	InF18 NDM-1-r	tagaggatcGAATTCTCAGCGCAGCTTGTCGGC
	InF18 KPC-2-f	ACAAAATCCATTCTCATGTCACTGTATCGCCGTC
	InF18 KPC-2-r	tagaggatcGAATTCTTACTGCCCGTTGACGCC
	InF18 VIM-2-f	ACAAAATCCATTCTCATGTTCAAACTTTTGAGTAAG
	InF18 VIM-2-r	tagaggatcGAATTCCTACTCAACGACTGAGCG
	InF18 IMP-1-f	ACAAAATCCATTCTCATGAGCAAGTTATCTGTATTC
	InF18 IMP-1-r	tagaggatcGAATTCTTAGTTGCTTGGTTTTGATG
	InF18 OXA-48-f	ACAAAATCCATTCTCATGCGTGTATTAGCCTTATC
	InF18 OXA-48-r	tagaggatcGAATTCCTAGGGAATAATTTTTTCCTG
	InF18 mcr-1-f	ACAAAATCCATTCTCATGATGCAGCATACTTCTG
	InF18 mcr-1-r	tagaggatcGAATTCTCAGCGGATGAATGCGGTG
	InF18 mcr-2-f	ACAAAATCCATTCTCATGACATCACATCACTCTTG
	InF18 mcr-2-r	tagaggatcGAATTCTTACTGGATAAATGCCGC
	InF18 RFP-f	ACAAAATCCATTCTCatggcgagtagcgaagac
	InF18 RFP-r	tagaggatcGAATTCttaagcaccggtggagtg
	InF pKLC26 for Hygro-f	GAATTCgatcctctagagtcg
	InF pKLC26 for Hygro-r	GAGAATGGATTTTGTGATGCC
	InF HygroR-f	ACAAAATCCATTCTCatgaaaaagcctgaactcac
	InF HygroR-r	tagaggatcGAATTCttattcctttgccctcggac
	InF42 HygroR-f	Aaggaggtacccaccatgaaaaagcctgaactcac
	InF42 HygroR-r	AAGCGATCGtctagatgcttcgtccatttgacag
	InF55 pKLC26IMP1-f	ctgcaggcatgcaagcttg
	InF55 pKLC26IMP1-r	gtcgactctagaggatcGAATTC
	InF55 pKLC26mcr2-f	tcctctagagtcgacACAAAATCCATTCTCATGACATCAC
	InF55 pKLC26mcr2-r	cttgcatgcctgcagGAATTCTTACTGGATAAATGCCG
	InF56 Tet-ind clo-f	GAACTCGAGCAGCTGAAGC
	InF56 Tet-ind clo-r for IMP-1	AGATAACTTGCTCATGGATCCtttctcctctttagatc
	InF56 pKLC26_IMP-1-f	ATGAGCAAGTTATCTGTATTCTTTA
	InF56 pKLC26_IMP1-mock-RFP-r	CAGCTGCTCGAGTTCtgcttcgtccatttgacag
	InF56 Tet-ind clo-r for mock	cgactctagaggatcGAATTCtttctcctctttagatc
	InF56 pKLC26_mock-f	gatcctctagagtcgacctg
	InF56 Tet-ind clo-r for RFP	ttcgctactcgccatGGATCCtttctcctctttagatc
	InF56 pKLC26_RFP-f	atggcgagtagcgaagacg
	RecA promoter oligo	tctagaCGATCGCTTGATAAGGTCCACGTAGCTGCTATAATTGCTTCAACAGAACATATTGA<br>CTATCCGGTATTACCCGGCAGATCTTTGTCGATCCTACCATCCACTCGACACACCCGCC<br>AGAATTCAAaggaggtacccacc
Oligo DNA sequences for crRNA in E. coli	crR-IMP-1_104-as	tatccATGTTCATACTTCGTTTGAAGAAGTTAA
	crR-IMP-1_104-s	aaacTTAACTTCTTCAAACGAAGTATGAACATg
	crR-IMP-1_291-as	tatccTAGCGACAGCACGGGCGGAATAGAGTGG
	crR-IMP-1_291-s	aaacCCACTCTATTCCGCCCGTGCTGTCGCTAg
	crR-IMP-1_298-as	tatccAGCACGGGCGGAATAGAGTGGCTTAATT
	crR-IMP-1_298-s	aaacAATTAAGCCACTCTATTCCGCCCGTGCTg

	crR-IMP-1_300-as	tatccCACGGGCGGAATAGAGTGGCTTAATTCT
	crR-IMP-1_300-s	aaacAGAATTAAGCCACTCTATTCCGCCCGTGg
	crR-IMP-1_331-as	tatccTCTATCCCCACGTATGCATCTGAATTAA
	crR-IMP-1_331-s	aaacTTAATTCAGATGCATACGTGGGGATAGAg
	crR-IMP-1_339-as	tatccCACGTATGCATCTGAATTAACAAATGAA
	crR-IMP-1_339-s	aaacTTCATTTGTTAATTCAGATGCATACGTGg
	crR-IMP-1_436-as	tatccAATAAAATTGAAGTTTTTTATCCAGGCC
	crR-IMP-1_436-s	aaacGGCCTGGATAAAAAACTTCAATTTTATTg
	crR-IMP-1_615-as	tatccTGGTAAGGCAAAACTGGTTGTTCCAAGT
	crR-IMP-1_615-s	aaacACTTGGAACAACCAGTTTTGCCTTACCAg
	crR-KPC-2_232-as	tatccGCTGTGCTGGCTCGCAGCCAGCAGCAGG
	crR-KPC-2_232-s	aaacCCTGCTGCTGGCTGCGAGCCAGCACAGCg
	crR-KPC-2_50-as	tatccTGGCTGGCTTTTCTGCCACCGCGCTGAC
	crR-KPC-2_50-s	aaacGTCAGCGCGGTGGCAGAAAAGCCAGCCAg
	crR-KPC-2_732-as	tatccTGACTATGCCGTCGTCTGGCCCACTGGG
	crR-KPC-2_732-s	aaacCCCAGTGGGCCAGACGACGGCATAGTCAg
	crR-NDM-1_117-as	tatccGGAAACTGGCGACCAACGGTTTGGCGAT
	crR-NDM-1_117-s	aaacATCGCCAAACCGTTGGTCGCCAGTTTCCg
	crR-NDM-1_406-as	tatccGGGATTGCGACTTATGCCAATGCGTTGT
	crR-NDM-1_406-s	aaacACAACGCATTGGCATAAGTCGCAATCCCg
	crR-NDM-1_724-as	tatccAAGGCCAGCATGATCGTGATGAGCCATT
	crR-NDM-1_724-s	aaacAATGGCTCATCACGATCATGCTGGCCTTg
	crR-OXA-48_201-as	tatccACCCGCATCTACCTTTAAAATTCCCAAT
	crR-OXA-48_201-s	aaacATTGGGAATTTTAAAGGTAGATGCGGGTg
	crR-OXA-48_364-as	tatccGTTTATCAAGAATTTGCCCGCCAAATTG
	crR-OXA-48_364-s	aaacCAATTTGGCGGGCAAATTCTTGATAAACg
	crR-OXA-48_470-as	tatccGGCTCGACGGTGGTATTCGAATTTCGGC
	crR-OXA-48_470-s	aaacGCCGAAATTCGAATACCACCGTCGAGCCg
	crR-VIM-2_229-as	tatccGGTGATGAGTTGCTTTTGATTGATACAG
	crR-VIM-2_229-s	aaacCTGTATCAATCAAAAGCAACTCATCACCg
	crR-VIM-2_335-as	tatccCCACGCACTTTCATGACGACCGCGTCGG
	crR-VIM-2_335-s	aaacCCGACGCGGTCGTCATGAAAGTGCGTGGg
	crR-VIM-2_518-as	tatccTCTATCCTGGTGCTGCGCATTCGACCGA
	crR-VIM-2_518-s	aaacTCGGTCGAATGCGCAGCACCAGGATAGAg
	crR-mcr-1_1021-as	tatccAAAGCGCAATTTGCCGATTATAAATCCG
	crR-mcr-1_1021-s	aaacCGGATTTATAATCGGCAAATTGCGCTTTg
	crR-mcr-1_1495-as	tatccGATAAGCAAACTGGCATCACGCCAATGG
	crR-mcr-1_1495-s	aaacCCATTGGCGTGATGCCAGTTTGCTTATCg
	crR-mcr-1_47-as	tatccTTGTTCTTGTGGCGAGTGTTGCCGTTTT
	crR-mcr-1_47-s	aaacAAAACGGCAACACTCGCCACAAGAACAAg
	crR-mcr-2_1015-as	tatccGCCACGCAGTATTTTGATTATAAATCAG
	crR-mcr-2_1015-s	aaacCTGATTTATAATCAAAATACTGCGTGGCg
	crR-mcr-2_1487-as	tatccCAAATAATACGACATTCAAGCCAACTGC
	crR-mcr-2_1487-s	aaacGCAGTTGGCTTGAATGTCGTATTATTTGg
	crR-mcr-2_37-as	tatccAATCCTTTTGTGCTGATGGGTTTGGTGG
	crR-mcr-2_37-s	aaacCCACCAAACCCATCAGCACAAAAGGATTg
	crR-stx1_640-as	tatccCACCGGAAGAAGTGGAACTCACACTGAA
	crR-stx1_640-s	aaacTTCAGTGTGAGTTCCACTTCTTCCGGTGg
	crR-stx2_640-as	tatccCGCCGGGAGACGTGGACCTCACTCTGAA
	crR-stx2_640-s	aaacTTCAGAGTGAGGTCCACGTCTCCCGGCGg
	Cas13 spacer IMP-1_563-s	aaacGACTTTGGCCAAGCTTCTATATTTGCGTg
	Cas13 spacer IMP-1_563-as	tatccACGCAAATATAGAAGCTTGGCCAAAGTC
	Cas9 spacer IMP-1_560-s	aaacGTGACGCAAATATAGAAGCTg
	Cas9 spacer IMP-1_560-as	aaaacAGCTTCTATATTTGCGTCAC
Oligo DNA sequences for crRNA in S. aureus	BsaI rpsE 40-as	tatccTAGAGAAGAAGAGACGAAAGAATTTGAAGAA
	BsaI rpsE 40-s	aaacTTCTTCAAATTCTTTCGTCTCTTCTTCTCTAg
	BsaI rpsE 65-as	tatccGAAGAACGCGTTGTTACAATCAACCGTGTAG
	BsaI rpsE 65-s	aaacCTACACGGTTGATTGTAACAACGCGTTCTTCg
	BsaI rpsE 85-as	tatccCAACCGTGTAGCAAAAGTTGTAAAAGGTGGT
	BsaI rpsE 85-s	aaacACCACCTTTTACAACTTTTGCTACACGGTTGg
	BsaI rpsE 104-as	tatccGTAAAAGGTGGTCGTCGTTTCCGTTTCACTG
	BsaI rpsE 104-s	aaacCAGTGAAACGGAAACGACGACCACCTTTTACg
	BsaI rpsE 133-as	tatccTGCATTAGTTGTAGTTGGAGACAAAAATGGT
	BsaI rpsE 133-s	aaacACCATTTTTGTCTCCAACTACAACTAATGCAg
	BsaI rpsE 158-as	tatccAATGGTCGTGTAGGTTTCGGTACTGGTAAAG
	BsaI rpsE 158-s	aaacCTTTACCAGTACCGAAACCTACACGACCATTg
	BsaI rpsE 176-as	tatccGGTACTGGTAAAGCTCAAGAGGTACCAGAAG
	BsaI rpsE 176-s	aaacCTTCTGGTACCTCTTGAGCTTTACCAGTACCg
	BsaI rpsE 197-as	tatccGTACCAGAAGCAATCAAAAAAGCTGTTGAAG
	BsaI rpsE 197-s	aaacCTTCAACAGCTTTTTTGATTGCTTCTGGTACg
	BsaI rpsE 223-as	tatccTGAAGCAGCTAAAAAAGATTTAGTAGTTGTT
	BsaI rpsE 223-s	aaacAACAACTACTAAATCTTTTTTAGCTGCTTCAg
	BsaI rpsE 242-as	tatccTTAGTAGTTGTTCCACGTGTTGAAGGTACAA
	BsaI rpsE 242-s	aaacTTGTACCTTCAACACGTGGAACAACTACTAAg
	BsaI rpsE 262-as	tatccTGAAGGTACAACTCCACACACAATTACTGGC
	BsaI rpsE 262-s	aaacGCCAGTAATTGTGTGTGGAGTTGTACCTTCAg
	BsaI rpsE 279-as	tatccACACAATTACTGGCCGTTACGGTTCAGGAAG
	BsaI rpsE 279-s	aaacCTTCCTGAACCGTAACGGCCAGTAATTGTGTg
	BsaI rpsE 308-as	tatccAGCGTATTTATGAAACCGGCTGCACCTGGTA

BsaI rpsE 308-s	aaacTACCAGGTGCAGCCGGTTTCATAAATACGCTg
BsaI rpsE 329-as	tatccGCACCTGGTACAGGAGTTATCGCTGGTGGTC
BsaI rpsE 329-s	aaacGACCACCAGCGATAACTCCTGTACCAGGTGCg
BsaI rpsE 353-as	tatccGGTGGTCCTGTTCGTGCCGTACTTGAATTAG
BsaI rpsE 353-s	aaacCTAATTCAAGTACGGCACGAACAGGACCACCg
BsaI rpsE 380-as	tatccTTAGCAGGTATCACTGATATCTTAAGTAAAT
BsaI rpsE 380-s	aaacATTTACTTAAGATATCAGTGATACCTGCTAAg
BsaI rpsE 398-as	tatccATCTTAAGTAAATCATTAGGATCAAACACAC
BsaI rpsE 398-s	aaacGTGTGTTTGATCCTAATGATTTACTTAAGATg
BsaI rpsE 423-as	tatccACACACCAATCAACATGGTTCGTGCTACAAT
BsaI rpsE 423-s	aaacATTGTAGCACGAACCATGTTGATTGGTGTGTg
BsaI rpsE 449-as	tatccACAATCGATGGTTTACAAAACCTTAAAAATG
BsaI rpsE 449-s	aaacCATTTTTAAGGTTTTGTAAACCATCGATTGTg
BsaI rpsE 474-as	tatccAAAATGCTGAAGATGTTGCGAAATTACGTGG
BsaI rpsE 474-s	aaacCCACGTAATTTCGCAACATCTTCAGCATTTTg
BsaI ermC 33-as	tatccTGAACGAGAAAAATATAAAACACAGTCAAAA
BsaI ermC 33-s	aaacTTTTGACTGTGTTTTATATTTTTCTCGTTCAg
BsaI ermC 71-as	tatccACTTCAAAACATAATATAGATAAAATAATGA
BsaI ermC 71-s	aaacTCATTATTTTATCTATATTATGTTTTGAAGTg
BsaI ermC 102-as	tatccCAAATATAAGATTAAATGAACATGATAATAT
BsaI ermC 102-s	aaacATATTATCATGTTCATTTAATCTTATATTTGg
BsaI ermC 134-as	tatccTTTGAAATCGGCTCAGGAAAAGGGCATTTTA
BsaI ermC 134-s	aaacTAAAATGCCCTTTTCCTGAGCCGATTTCAAAg
BsaI ermC 167-as	tatccCTTGAATTAGTACAGAGGTGTAATTTCGTAA
BsaI ermC 167-s	aaacTTACGAAATTACACCTCTGTACTAATTCAAGg
BsaI ermC 200-as	tatccGCCATTGAAATAGACCATAAATTATGCAAAA
BsaI ermC 200-s	aaacTTTTGCATAATTTATGGTCTATTTCAATGGCg
BsaI ermC 234-as	tatccCAGAAAATAAACTTGTTGATCACGATAATTT
BsaI ermC 234-s	aaacAAATTATCGTGATCAACAAGTTTATTTTCTGg
BsaI ermC 272-as	tatccTTAAACAAGGATATATTGCAGTTTAAATTTC
BsaI ermC 272-s	aaacGAAATTTAAACTGCAATATATCCTTGTTTAAg
BsaI ermC 307-as	tatccAAACCAATCCTATAAAATATTTGGTAATATA
BsaI ermC 307-s	aaacTATATTACCAAATATTTTATAGGATTGGTTTg
BsaI ermC 336-as	tatccTACCTTATAACATAAGTACGGATATAATACG
BsaI ermC 336-s	aaacCGTATTATATCCGTACTTATGTTATAAGGTAg
BsaI ermC 378-as	tatccTTGATAGTATAGCTGATGAGATTTATTTAAT
BsaI ermC 378-s	aaacATTAAATAAATCTCATCAGCTATACTATCAAg
BsaI ermC 418-as	tatccCGAGTTTGCTAAAAGATTATTAAATACAAAA
BsaI ermC 418-s	aaacTTTTGTATTTAATAATCTTTTAGCAAACTCGg
BsaI ermC 458-as	tatccGCATTATTTTTAATGGCAGAAGTTGATATTT
BsaI ermC 458-s	aaacAAATATCAACTTCTGCCATTAAAAATAATGCg
BsaI ermC 490-as	tatccTATATTAAGTATGGTTCCAAGAGAATATTTT
BsaI ermC 490-s	aaacAAAATATTCTCTTGGAACCATACTTAATATAg
BsaI ermC 522-as	tatccATCCTAAACCTAAAGTGAATAGCTCACTTAT
BsaI ermC 522-s	aaacATAAGTGAGCTATTCACTTTAGGTTTAGGATg
BsaI ermC 565-as	tatccAAAAAAATCAAGAATATCACACAAAGATAAA
BsaI ermC 565-s	aaacTTTATCTTTGTGTGATATTCTTGATTTTTTTg
BsaI ermC 600-as	tatccAGTATAATTATTTCGTTATGAAATGGGTTAA
BsaI ermC 600-s	aaacTTAACCCATTTCATAACGAAATAATTATACTg
BsaI ermC 639-as	tatccACAAGAAAATATTTACAAAAAATCAATTTAA
BsaI ermC 639-s	aaacTTAAATTGATTTTTTGTAAATATTTTCTTGTg
BsaI ermC 675-as	tatccCCTTAAAACATGCAGGAATTGACGATTTAAA
BsaI ermC 675-s	aaacTTTAAATCGTCAATTCCTGCATGTTTTAAGGg
BsaI ermC 714-as	tatccGCTTTGAACAATTCTTATCTCTTTTCAATAG
BsaI ermC 714-s	aaacCTATTGAAAAGAGATAAGAATTGTTCAAAGCg
BsaI MecA 48-as	tatccTTGTTCCACTTATTTTAATAGTTGTAGTTGT
BsaI MecA 48-s	aaacACAACTACAACTATTAAAATAAGTGGAACAAg
BsaI MecA 147-as	tatccATAAAAATTTCAAACAAGTTTATAAAGATAG
BsaI MecA 147-s	aaacCTATCTTTATAAACTTGTTTGAAATTTTTATg
BsaI MecA 247-as	tatccTTTAGGCGTTAAAGATATAAACATTCAGGAT
BsaI MecA 247-s	aaacATCCTGAATGTTTATATCTTTAACGCCTAAAg
BsaI MecA 337-as	tatccAACAAACTACGGTAACATTGATCGCAACGTT
BsaI MecA 337-s	aaacAACGTTGCGATCAATGTTACCGTAGTTTGTTg
BsaI MecA 446-as	tatccGACCAAAGCATACATATTGAAAATTTAAAAT
BsaI MecA 446-s	aaacATTTTAAATTTTCAATATGTATGCTTTGGTCg
BsaI MecA 554-as	tatccCCAAAGAATGTATCTAAAAAAGATTATAAAG
BsaI MecA 554-s	aaacCTTTATAATCTTTTTTAGATACATTCTTTGGg
BsaI MecA 648-as	tatccTACAAGATGATACCTTCGTTCCACTTAAAAC
BsaI MecA 648-s	aaacGTTTTAAGTGGAACGAAGGTATCATCTTGTAg
BsaI MecA 749-as	tatccAGTCGTAACTATCCTCTAGGAAAAGCGACTT
BsaI MecA 749-s	aaacAAGTCGCTTTTCCTAGAGGATAGTTACGACTg
BsaI MecA 849-as	tatccAAGATGATGCAGTTATTGGTAAAAAGGGACT
BsaI MecA 849-s	aaacAGTCCCTTTTTACCAATAACTGCATCATCTTg
BsaI MecA 963-as	tatccATACATTAATAGAGAAAAAGAAAAAGATGG
BsaI MecA 963-s	aaacCCATCTTTTTCTTTTTCTCTATTAATGTATg
BsaI MecA 1050-as	tatccTGAAAAATGATTATGGCTCAGGTACTGCTAT
BsaI MecA 1050-s	aaacATAGCAGTACCTGAGCCATAATCATTTTTCAg
BsaI MecA 1151-as	tatccGGCATGAGTAACGAAGAATATAATAAATTAA
BsaI MecA 1151-s	aaacTTAATTTATTATATTCTTCGTTACTCATGCCg

	BsaI MecA 1248-as	tatccAAATATTAACAGCAATGATTGGGTTAAATAA
	BsaI MecA 1248-s	aaacTTATTTAACCCAATCATTGCTGTTAATATTTg
	BsaI MecA 1359-as	tatccTTACAAGATATGAAGTGGTAAATGGTAATAT
	BsaI MecA 1359-s	aaacATATTACCATTTACCACTTCATATCTTGTAAg
	BsaI MecA 1446-as	tatccTCGAATTAGGCAGTAAGAAATTTGAAAAAGG
	BsaI MecA 1446-s	aaacCCTTTTTCAAATTTCTTACTGCCTAATTCGAg
	BsaI MecA 1550-as	tatccAATTTAGATAATGAAATATTATTAGCTGATT
	BsaI MecA 1550-s	aaacAATCAGCTAATAATATTTCATTATCTAAATTg
	BsaI MecA 1644-as	tatccTAGAAAATAATGGCAATATTAACGCACCTCA
	BsaI MecA 1644-s	aaacTGAGGTGCGTTAATATTGCCATTATTTTCTAg
	BsaI MecA 1747-as	tatccTGATGGTATGCAACAAGTCGTAAATAAAACA
	BsaI MecA 1747-s	aaacTGTTTTATTTACGACTTGTTGCATACCATCAg
	BsaI MecA 1856-as	tatccGAAACTGGCAGACAAATTGGGTGGTTTATAT
	BsaI MecA 1856-s	aaacATATAAACCACCCAATTTGTCTGCCAGTTTCg
	BsaI MecA 1946-as	tatccAAAGGAATGGCTAGCTACAATGCCAAAATCT
	BsaI MecA 1946-s	aaacAGATTTTGGCATTGTAGCTAGCCATTCCTTTg
Construction of pKLC31	InFpi araCosPPi NotI1-f	cttctccatgcggccatcgatgcataatgtgcctg
	InFpi araCosPPi NotI1-r	agcctaccggcggccTGCTGCGAGTGATTAACAAG
Construction of pKLC27	InF27 araCosPPi-f	GGCAATTCCGACGTCatcgatgcataatgtgcctg
	InF27 araCosPPi-r	TCAAAACGCGTCGACttactgttctgtatcggctg
	InF27 pRC319-f	gtcgacgcgttttgaaagc
	InF27 pRC319-r	gacgtcggaattgccagctg
Construction of antibiotics resistant gene expression vector	pKLC26-f	GAATTCgatcctctagagtcg
	pKLC26-r	GAGAATGGATTTTGTGATGCC
	HygroR-f	ACAAAATCCATTCTCatgaaaaagcctgaactcac
	HygroR-r	tagaggatcGAATTCttattcctttgccctcggac
	pAH143 GenR-f	ACAAAATCCATTCTCatgttatggagcagcaacga
	pAH143 GenR-r	tagaggatcGAATTCttaggtggcggtacttgggt
	pAH144 SpcR-StrR-f	ACAAAATCCATTCTCatgcgctcacgcaactggtc
	pAH144 SpcR-StrR-r	tagaggatcGAATTCttatttgccgactaccttgg
	pAH145 TmpR-f	ACAAAATCCATTCTCatgggtcaaagtagcgatga
	pAH145 TmpR-r	tagaggatcGAATTCttaggccacacgttcaagtg
	pRTKH2 ErmR-f	ACAAAATCCATTCTCatgaacaaaaatataaaatattctc
	pRTKH2 ErmR-r	tagaggatcGAATTCttatttcctcccgttaaataatag
	PuroR-f	ACAAAATCCATTCTCggtgcgcctcgccacccg
	PuroR-r	tagaggatcGAATTCtcaggcaccgggcttgcg
Construction of pKLC44	InF44 pKLC42-f	cagggcgcgtctcgaGtctagaCGATCGCTTGATAAGG
	InF44 pKLC42-r	acaggagtccaagcgAGCTcgaaagcaaattcgCTCGAG
Genetic modification of E. coli	K12 genome-in pKLC26-s	cccttcaaccttagcagtagcgtgggatgatttcacaattagaaagacctTGACGCACACCGTGGAAAC
	K12 genome-in pKLC26Cm-as	tgtcctgcacgacgccttgcgtcactagcctcttctctagctcatcatgcTGAGACGTTGATCGGCACG
	K12 genome-in pKLC23_26_56-s	cccttcaaccttagcagtagcgtgggatgatttcacaattagaaagacctGCCTGTCAAATGGACGAAGC
	K12 genome-in pKLC23_26_56Cm-as	tgtcctgcacgacgccttgcgtcactagcctcttctctagctcatcatgcTGAGACGTTGATCGGCACG
Detection primers for S. aureus	SA 23S rRNA det-s	AAGCGAGTCTGAATAGGGCG
	SA 23S rRNA det-as	AACGTAAGTCGGTTCGGTCC
	SA RPF3757 MecA det-s	TGGCTCAGGTACTGCTATCC
	SA RPF3757 MecA det-as	AGACGTCATATGAAGGTGTGC
	SA RPF3757 ermC det-s	CTTTGAAATCGGCTCAGGAAAAGG
	SA RPF3757 ermC det-as	GGCAGTTACGAAATTACACCTCTG
	SA RPF3757 rpsE det-s	GGTCGTCGTTTCCGTTTCAC
	SA RPF3757 rpsE det-as	TGCTTCTGGTACCTCTTGAGC
Construction of target expression vectors for S. aureus	USA300p3 ermC clo BamHI-s	ATATGGATCCATGAACGAGAAAAATATAAAACACAG
	USA300p3 ermC clo EcoRI-as	ATATGAATTCCAAAAGACATAATCGATTCAC
	rpsE clo BamHI-s	atatGGATCCatggctcgtagagaagaag
	rpsE clo SacI-as	atatGAGCTCcctccttaattgtataattcttctac
	USA300g MecA clo BamHI-s2	ATATGGATCCGTAGTCTTATATAAGGAGTATATTG
	USA300g MecA clo EcoRI-as2	ATATGAATTCTAAGGGAGAAGTAACAGCAC
	RFP clo BamHI-s	ATATGGATCCATGGCGAGTAGCGAAGAC
	RFP clo EcoRI-as	ATATGAATTCTTAAGCACCGGTGGAGTG
Construction of stx1/2 expression vector	pKLC26 stx1 partial PCR SacI-as	GTGGAGCTCGGTCATGGCATTTCCACTAAACTCCATTAAGAGAATGGATTTTGTGATGC
	pKLC26 stx1 partial PCR SacI -s	ACCGAGCTCCACCGGAAGAAGTGGAACTCACACTGAACGAATTCgatcctctagagtc
	pKLC26 stx2 partial PCR SacI -as	GCGGAGCTCGGTCATTGTATTACCACTGAACTCCATTAAGAGAATGGATTTTGTGATGC
	pKLC26 stx2 partial PCR SacI -s	ACCGAGCTCCGCCGGGAGACGTGGACCTCACTCTGAACGAATTCgatcctctagagtc
Construction of SaPI-based CapsidCas13a plasmid	BAPup 7272-F	AATTCCTGCAGCCCGTTGACGAGGTTGGTAATGGCAC
	BAPup 8172-R	CCCTGTTGATACCGGGATACATCACTTGTTTTGCCGTC
	BAPdown 12224-F	TCGGCGCAAAGTGCGGGGCTCTCCACTTACAAAGGT
	BAPdown 13089-R	TTTGCCGTTACGCACGGGCACTCTTTTGTGTATAACCG
	LsC2c2 mecA5-F	CCGGTATCAACAGGGATGCCTACAGCATCCAGGGT
	LsC2c2 mecA5-R	CGCACTTTGCGCCGAGAACCTTCGAAAAACCGCCC
	LsC2c2 188-R	CGCACTTTGCGCCGACCCATCCTAATTTCTTGCTGATGAG
Construction of pIMAY mecA deletion construct	USA300-C02 906894-F	AATTCCTGCAGCCCGCGCCCAAAGCTTCTTTAGCTG
	USA300-C02 907924-R	CCCTGTTGATACCGGTGTGATATGGAGGTGTAGAAGGTG
	USA300-C02 910027-F	TCGGCGCAAAGTGCGCCGTAACGATGGTTGCTTCAC
	USA300-C02 910980-R	TTTGCCGTTACGCACCTGGACCGAATGGACTAGCA
Detection primers for PCR	IMP-1 variants det-F	AAGGCGTTTATGTTCATACTTCG
	IMP-1 variants det-R	TTTAACCGCCTGCTCTAATGTAA
	blaNDM-1 420-F	TTGCCCAATATTATGCACCC
	blaNDM-1 420-R	ATTGGCATAAGTCGCAATCC
	Ec 16SrRNA 123 det-s	AAGGCCTTCGGGTTGTAAAG
	Ec 16SrRNA 123 det-as	CTTGCACCCTCCGTATTACC

	Cas13a det1-f	agtaaggatagatggggattatgag
	Cas13a det1-r	agatttctttttatcaccaccaac
	Cas13a det2-f	agtaatttagaaattctgggttttg
	Cas13a det2-r	ttctcgatctcctccaaaga
	Cas13a det3-f	tcacgctaaagaggaattgg
	Cas13a det3-r	accgcattactatttaacagtgc
	Cas13a det4-f	acgaaagaataactgtaaagactagtg
	Cas13a det4-r	agccatttgaatagcaagtttcc
	Cas13a det5-f	tggatattctaaagaatacaaagagaag
	Cas13a det5-r	tagaaccaccctatactattatacc
	Cas9 det1-f	catcctgtggagcttagtagg
	Cas9 det1-r	gctaaggccaaatagattaagc
	Cas9 det2-f	gtagatgaagttgcttatcatgag
	Cas9 det2-r	tccatgcaaaacgactattgc
	Cas9 det3-f	ctgcatgctattttgagaagac
	Cas9 det3-r	gagttgtctgattttcacgtg
	Cas9 det4-f	tgtaaaagttgttgatgaattggtc
	Cas9 det4-r	gacaatactttgcgcactgtg
	Cas9 det5-f	acttgcaaatggagagattcg
	Cas9 det5-r	gctagacttcagtcttgaaaagc
	IMP-1 53-56-70-71 cloning primer-f	ggatctgctcatgtttgacagc
	IMP-1 53-56 cloning primer-r	atcagaccgcttctgcgttc
	Cas13a spacer detection-f	gatacgttataagaatgaGatgggag
	Cas13a spacer detection-r	aggctctagttagcctaatcg
	Cas9 spacer detection-f	cagctaggaggtgactgaag
	Cas9 spacer detection-r	ctgcagcgatggatatgttc

Table S4. List of depletion rate of sequence reads

Position in IMP-1	Spacer sequence	Depletion rate	Survival rate of *E. coli* IMP-1	Sequence reads from *E. coli* mock				Sequence reads from *E. coli* IMP-1			
				Rep.1	Rep.2	Rep.3	Rep.4	Rep.1	Rep.2	Rep.3	Rep.4
IMP-1_563	GACTTTGGCCAAGCTTCTATATTTGCGT	99.66	0.34	56	75	47	67	1	0	3	0
IMP-1_702	TTTTGATGGTTTTTTACTTTCGTTTAAC	99.61	0.39	40	106	65	68	0	2	5	0
IMP-1_562	ACTTTGGCCAAGCTTCTATATTTGCGTC	99.60	0.40	44	88	58	70	0	4	3	0
IMP-1_370	TGGCTTGAACCTTACCGTCTTTTTTAAG	99.55	0.45	53	89	72	57	0	1	7	0
IMP-1_566	GCGGACTTTGGCCAAGCTTCTATATTTG	99.55	0.45	49	70	40	57	1	1	3	0
IMP-1_274	TGCTGTCGCTATGAAAATGAGAGGAAAT	99.37	0.63	42	52	58	67	0	1	6	2
IMP-1_565	CGGACTTTGGCCAAGCTTCTATATTTGC	99.36	0.64	55	80	55	46	2	0	3	3
IMP-1_193	TATCTTTAGCCGTAAATGGAGTGTCAAT	99.31	0.69	49	65	49	56	1	1	3	4
IMP-1_648	TTTCAAGAGTGATGCGTCTCCAACTTCA	99.27	0.73	47	91	60	54	0	2	6	3
IMP-1_11	CAAAACAAAAATATAAAGAATACAGATA	99.20	0.80	40	66	46	43	1	1	5	2
IMP-1_703	GTTTTGATGGTTTTTTACTTTCGTTTAA	99.15	0.85	54	69	69	51	2	2	3	5
IMP-1_208	TGACTAACTTTTCAGTATCTTTAGCCGT	99.09	0.91	46	65	55	49	0	3	4	5
IMP-1_609	AACAACCAGTTTTGCCTTACCATATTTG	99.00	1.00	41	67	46	74	4	1	6	0
IMP-1_206	ACTAACTTTTCAGTATCTTTAGCCGTAA	98.96	1.04	48	67	54	49	5	0	7	1
IMP-1_322	ATGCATACGTGGGGATAGATCGAGAATT	98.95	1.05	49	75	56	55	3	1	6	4
IMP-1_181	TAAATGGAGTGTCAATTAGGTAAGCCTC	98.90	1.10	35	41	49	46	5	0	5	1
IMP-1_339	TTCATTTGTTAATTCAGATGCATACGTG	98.89	1.11	30	103	50	52	1	4	6	4
IMP-1_368	GCTTGAACCTTACCGTCTTTTTTAAGCA	98.89	1.11	62	77	56	54	3	6	6	2
IMP-1_637	ATGCGTCTCCAACTTCACTGTGACTTGG	98.88	1.12	39	36	36	38	2	3	4	1
IMP-1_269	TCGCTATGAAAATGAGAGGAAATGCTGC	98.86	1.14	39	22	23	25	4	0	4	0
IMP-1_170	TCAATTAGGTAAGCCTCAGCATTTACAA	98.78	1.22	11	84	26	53	0	12	3	2
IMP-1_652	TAAGTTTCAAGAGTGATGCGTCTCCAAC	98.78	1.22	43	62	56	57	6	2	6	1
IMP-1_363	AACCTTACCGTCTTTTTTAAGCAGTTCA	98.65	1.35	37	81	68	55	2	8	12	0
IMP-1_272	CTGTCGCTATGAAAATGAGAGGAAATGC	98.48	1.52	38	75	41	40	5	3	3	5
IMP-1_23	GTAGCAATGCTGCAAAACAAAAATATAA	98.48	1.52	47	57	61	42	3	7	4	5
IMP-1_471	CAACCAAACCACTACGTTATCTGGAGTG	98.43	1.57	42	73	50	54	6	9	4	2
IMP-1_615	ACTTGGAACAACCAGTTTTGCCTTACCA	98.33	1.67	28	39	26	32	3	3	5	1
IMP-1_376	AATTTGTGGCTTGAACCTTACCGTCTTT	98.31	1.69	40	93	59	56	9	3	6	3
IMP-1_120	AACAACGCCCCACCCGTTAACTTCTTCA	98.26	1.74	43	70	54	64	10	3	8	0
IMP-1_121	GAACAACGCCCCACCCGTTAACTTCTTC	98.21	1.79	39	65	43	49	5	14	4	0
IMP-1_342	CAGTTCATTTGTTAATTCAGATGCATAC	98.20	1.80	12	88	31	51	1	13	4	4
IMP-1_472	GCAACCAAACCACTACGTTATCTGGAGT	98.14	1.86	44	70	46	80	2	8	10	6
IMP-1_104	TTAACTTCTTCAAACGAAGTATGAACAT	98.12	1.88	17	58	29	40	3	6	3	3
IMP-1_639	TGATGCGTCTCCAACTTCACTGTGACTT	97.97	2.03	26	31	33	26	6	2	4	2
IMP-1_448	GAGTGTGTCCCGGGCCTGGATAAAAAAC	97.93	2.07	50	80	51	63	4	11	13	2
IMP-1_266	CTATGAAAATGAGAGGAAATGCTGCCTT	97.86	2.14	8	31	10	32	1	2	3	1
IMP-1_81	AACATAAACGCCTTCATCAAGCTTTTCA	97.72	2.28	49	76	57	52	5	10	14	4
IMP-1_555	CCAAGCTTCTATATTTGCGTCACCCAAA	97.68	2.32	45	78	53	49	12	5	7	5
IMP-1_392	TTAACTCCGCTAAATGAATTTGTGGCTT	97.68	2.32	11	25	7	22	0	0	3	3
IMP-1_610	GAACAACCAGTTTTGCCTTACCATATTT	97.61	2.39	35	62	61	41	9	10	7	2
IMP-1_550	CTTCTATATTTGCGTCACCCAAATTGCC	97.57	2.43	57	72	46	49	7	6	13	5
IMP-1_28	CTGCGGTAGCAATGCTGCAAAACAAAAA	97.16	2.84	53	104	64	57	11	6	15	11
IMP-1_600	TTTTGCCTTACCATATTTGGACTTTAAT	97.11	2.89	51	66	38	54	12	8	9	6
IMP-1_367	CTTGAACCTTACCGTCTTTTTTAAGCAG	97.08	2.92	54	94	41	81	10	16	11	7
IMP-1_660	CTCTAATGTAAGTTTCAAGAGTGATGCG	96.82	3.18	45	71	61	47	10	15	13	6
IMP-1_88	AAGTATGAACATAAACGCCTTCATCAAG	96.72	3.28	54	70	49	51	7	13	17	7
IMP-1_157	CCTCAGCATTTACAAGAACCACCAAACC	96.53	3.47	56	31	71	16	30	0	22	0
IMP-1_126	TTTAGGAACAACGCCCCACCCGTTAACT	96.37	3.63	39	62	62	71	16	9	16	5
IMP-1_697	ATGGTTTTTTACTTTCGTTTAACCCTTT	96.30	3.70	30	59	46	46	8	13	9	10
IMP-1_60	CTTTTCAATTTTTAAATCTGGCAAAGAC	95.93	4.07	35	83	39	41	10	13	10	12
IMP-1_250	AAATGCTGCCTTTTATTTTATAGCCACG	95.85	4.15	13	24	19	15	2	6	9	2
IMP-1_162	GTAAGCCTCAGCATTTACAAGAACCACC	95.74	4.26	54	53	52	42	20	15	20	0
IMP-1_141	AACCACCAAACCATGTTTAGGAACAACG	95.47	4.53	31	54	35	51	11	16	11	7
IMP-1_85	TATGAACATAAACGCCTTCATCAAGCTT	95.30	4.70	20	67	26	47	6	22	4	17
IMP-1_556	GCCAAGCTTCTATATTTGCGTCACCCAA	95.23	4.77	46	63	37	52	8	17	18	12
IMP-1_383	CTAAATGAATTTGTGGCTTGAACCTTAC	95.21	4.79	38	72	79	51	24	15	15	7
IMP-1_710	TTGCTTGGTTTTGATGGTTTTTTACTTT	95.13	4.87	34	68	41	49	15	18	8	14
IMP-1_227	CCACGCTCCACAAACCAAGTGACTAACT	95.12	4.88	37	86	58	44	22	13	19	5
IMP-1_184	CCGTAAATGGAGTGTCAATTAGGTAAGC	94.63	5.37	50	63	32	46	8	12	23	11
IMP-1_617	TGACTTGGAACAACCAGTTTTGCCTTAC	94.46	5.54	37	69	36	60	14	21	12	20
IMP-1_632	TCTCCAACTTCACTGTGACTTGGAACAA	93.98	6.02	42	82	48	60	26	23	16	14
IMP-1_19	CAATGCTGCAAAACAAAAATATAAAGAA	93.96	6.04	55	74	59	75	28	34	21	11
IMP-1_680	TTTAACCCTTTAACCGCCTGCTCTAATG	93.74	6.26	43	97	40	64	28	20	21	9
IMP-1_353	TCTTTTTTAAGCAGTTCATTTGTTAATT	93.56	6.44	35	71	52	48	20	21	17	18
IMP-1_447	AGTGTGTCCCGGGCCTGGATAAAAAACT	93.53	6.47	44	109	67	71	31	30	20	21
IMP-1_34	ACTCTGCTGCGGTAGCAATGCTGCAAAA	93.39	6.61	51	87	57	56	30	26	28	13
IMP-1_161	TAAGCCTCAGCATTTACAAGAACCACCA	93.23	6.77	30	69	42	52	24	21	14	11
IMP-1_445	TGTGTCCCGGGCCTGGATAAAAAACTTC	93.20	6.80	16	93	23	62	7	58	4	26
IMP-1_536	TCACCCAAATTGCCTAAACCGTACGGTT	93.13	6.87	31	63	33	42	15	26	13	16

IMP-1_261	AAAATGAGAGGAAATGCTGCCTTTTATT	92.95	7.05	3	53	6	25	2	10	3	9
IMP-1_251	GAAATGCTGCCTTTTATTTTATAGCCAC	91.76	8.24	11	19	4	17	4	8	3	8
IMP-1_664	CCTGCTCTAATGTAAGTTTCAAGAGTGA	91.38	8.62	39	75	53	42	33	25	31	14
IMP-1_39	CAAAGACTCTGCTGCGGTAGCAATGCTG	91.22	8.78	29	58	37	38	29	24	18	9
IMP-1_22	TAGCAATGCTGCAAAACAAAAATATAAA	90.97	9.03	51	71	61	45	27	45	32	23
IMP-1_580	ACTTTAATAATTTGGCGGACTTTGGCCA	90.47	9.53	41	71	62	68	30	43	39	24
IMP-1_174	AGTGTCAATTAGGTAAGCCTCAGCATTT	90.35	9.65	36	71	44	51	38	35	18	20
IMP-1_291	CCACTCTATTCCGCCCGTGCTGTCGCTA	89.79	10.21	4	23	9	13	5	7	7	2
IMP-1_25	CGGTAGCAATGCTGCAAAACAAAAATAT	89.07	10.93	28	71	33	41	30	30	20	23
IMP-1_430	GATAAAAAACTTCAATTTTATTTTTAAC	87.91	12.09	30	83	67	50	47	36	35	21
IMP-1_399	CCAATAGTTAACTCCGCTAAATGAATTT	86.88	13.12	38	36	54	17	36	20	31	19
IMP-1_511	GTTTAATAAAACAACCACCGAATAATAT	86.40	13.60	39	55	22	43	22	58	27	20
IMP-1_222	CTCCACAAACCAAGTGACTAACTTTTCA	84.00	16.00	53	85	78	46	79	89	50	33
IMP-1_641	AGTGATGCGTCTCCAACTTCACTGTGAC	83.97	16.03	18	24	18	12	19	15	25	10
IMP-1_172	TGTCAATTAGGTAAGCCTCAGCATTTAC	82.15	17.85	44	90	44	47	58	81	44	53
IMP-1_595	CCTTACCATATTTGGACTTTAATAATTT	81.73	18.27	42	74	53	45	49	86	57	47
IMP-1_151	CATTTACAAGAACCACCAAACCATGTTT	80.70	19.30	44	63	45	54	72	77	52	37
IMP-1_175	GAGTGTCAATTAGGTAAGCCTCAGCATT	80.60	19.40	23	30	32	23	46	29	27	21
IMP-1_310	GGATAGATCGAGAATTAAGCCACTCTAT	80.53	19.47	34	74	47	47	53	78	53	47
IMP-1_436	GGCCTGGATAAAAAACTTCAATTTTATT	80.06	19.94	30	42	29	19	38	46	25	31
IMP-1_429	ATAAAAAACTTCAATTTTATTTTTAACT	78.71	21.29	17	48	33	32	28	62	39	34
IMP-1_40	GCAAAGACTCTGCTGCGGTAGCAATGCT	78.67	21.33	3	81	22	57	13	12	11	12
IMP-1_309	GATAGATCGAGAATTAAGCCACTCTATT	78.49	21.51	44	63	51	34	61	74	63	49
IMP-1_538	CGTCACCCAAATTGCCTAAACCGTACGG	77.76	22.24	44	76	52	52	68	107	53	75
IMP-1_707	CTTGGTTTTGATGGTTTTTTACTTTCGT	76.18	23.82	52	83	54	67	110	101	76	71
IMP-1_435	GCCTGGATAAAAAACTTCAATTTTATTT	75.53	24.47	36	51	48	41	78	81	53	45
IMP-1_298	AATTAAGCCACTCTATTCCGCCCGTGCT	74.77	25.23	13	17	13	16	23	28	20	19
IMP-1_311	GGGATAGATCGAGAATTAAGCCACTCTA	74.45	25.55	43	59	61	42	76	87	67	79
IMP-1_113	CCCCACCCGTTAACTTCTTCAAACGAAG	74.22	25.78	42	72	52	56	100	109	57	72
IMP-1_308	ATAGATCGAGAATTAAGCCACTCTATTC	71.10	28.90	35	52	47	55	83	120	57	63
IMP-1_510	TTTAATAAAACAACCACCGAATAATATT	69.60	30.40	44	83	51	50	101	151	85	81
IMP-1_634	CGTCTCCAACTTCACTGTGACTTGGAAC	69.34	30.66	41	64	44	41	93	110	80	68
IMP-1_300	AGAATTAAGCCACTCTATTCCGCCCGTG	69.07	30.93	15	26	24	28	41	63	39	21
IMP-1_304	ATCGAGAATTAAGCCACTCTATTCCGCC	67.65	32.35	32	62	33	51	75	122	73	69
IMP-1_693	TTTTTTACTTTCGTTTAACCCTTTAACC	66.06	33.94	53	52	51	44	115	137	93	72
IMP-1_581	GACTTTAATAATTTGGCGGACTTTGGCC	64.66	35.34	42	96	46	92	146	198	94	94
IMP-1_245	CTGCCTTTTATTTTATAGCCACGCTCCA	62.79	37.21	11	43	24	25	52	64	26	44
IMP-1_278	CCCGTGCTGTCGCTATGAAAATGAGAGG	61.78	38.22	42	81	33	50	104	167	94	96
IMP-1_331	TTAATTCAGATGCATACGTGGGGATAGA	58.31	41.69	28	46	16	18	86	80	41	50
IMP-1_694	GTTTTTTACTTTCGTTTAACCCTTTAAC	57.28	42.72	30	54	38	49	113	147	82	86
IMP-1_455	TTATCTGGAGTGTGTCCCGGGCCTGGAT	52.86	47.14	40	44	63	41	109	178	109	122
IMP-1_443	TGTCCCGGGCCTGGATAAAAAACTTCAA	50.57	49.43	63	72	63	55	181	249	144	188
IMP-1_491	AATAATATTTTCCTTTCAGGCAACCAAA	48.06	51.94	44	27	53	21	217	71	161	43
IMP-1_242	CCTTTTATTTTATAGCCACGCTCCACAA	47.42	52.58	15	26	23	23	78	88	57	40
IMP-1_14	CTGCAAAACAAAAATATAAAGAATACAG	45.19	54.81	17	44	24	35	55	205	54	112
IMP-1_441	TCCCGGGCCTGGATAAAAAACTTCAATT	40.44	59.56	39	81	29	56	152	263	131	159
IMP-1_515	TACGGTTTAATAAAACAACCACCGAATA	37.33	62.67	48	92	67	68	260	338	222	194
IMP-1_524	CCTAAACCGTACGGTTTAATAAAACAAC	15.54	84.46	50	71	59	56	295	455	210	263
IMP-1_434	CCTGGATAAAAAACTTCAATTTTATTTT	9.39	90.61	26	84	55	46	243	338	209	225
IMP-1_71	CCTTCATCAAGCTTTTCAATTTTTAAAT	-0.01	100.01	46	58	51	39	321	364	252	240
IMP-1_253	AGGAAATGCTGCCTTTTATTTTATAGCC	-	-	0	6	4	5	4	1	3	7
IMP-1_255	AGAGGAAATGCTGCCTTTTATTTTATAG	-	-	6	2	6	0	13	2	6	6
NC	GGAGACCGAGATTGGTCTC	0.00	100.00	45	92	58	74	380	574	254	391
